# Metal Halide Perovskite/Chalcohalide Heterojunctions for the Photoinduced Oxidative Coupling of *p*‑Substituted Thiophenols

**DOI:** 10.1021/acsanm.5c05834

**Published:** 2026-03-06

**Authors:** Anna Cabona, Stefano Toso, Alejandro Cortés-Villena, Ignacio Rosa-Pardo, Mirko Prato, Michele Ferri, Julia Pérez-Prieto, Ilka Kriegel, Liberato Manna, Raquel E. Galian

**Affiliations:** † Nanochemistry Department, Italian Institute of Technology, 16163 Genova, Italy; ‡ Department of Applied Science and Technology, 19032 Politecnico di Torino, 10129 Turin, Italy; § Lund University, Division of Chemical Physics, Naturvetarvägen 14, 221 00 Lund, Sweden; ∥ Institute of Molecular Science, 16781 University of Valencia, c/Catedrático José Beltrán Martínez 2, 46980 Paterna, Valencia, Spain; ⊥ Materials Characterization, Italian Institute of Technology, 16163 Genova, Italy

**Keywords:** heterostructure, photocatalysis, heterojunction, perovskite, chalcohalide

## Abstract

The introduction of a semiconductor–semiconductor junction is an effective strategy to enhance the photocatalytic performance of perovskite nanocrystal-based systems. Herein, we optimized the synthesis of CsPbX_3_/Pb_4_S_3_Y_2_ (X, Y = Cl, Br, I) perovskite/chalcohalide heterostructures, whose band alignment can be tuned by the halide composition. As a proof-of-concept, we evaluated the photooxidative coupling of *p*-substituted thiophenols at room temperature, under visible light, in air, and without a sacrificial electron donor. Notably, CsPbBr_3_/Pb_4_S_3_Br_2_ achieved up to 94% selectivity toward disulfide (*p*-OCH_3_ thiophenol with a turnover number of 14 300), highlighting the crucial role of the type-II heterojunction in promoting charge separation and efficient electron delocalization across the junction.

## Graphical abstract

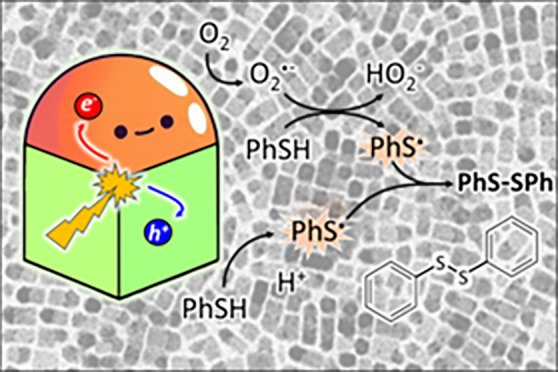

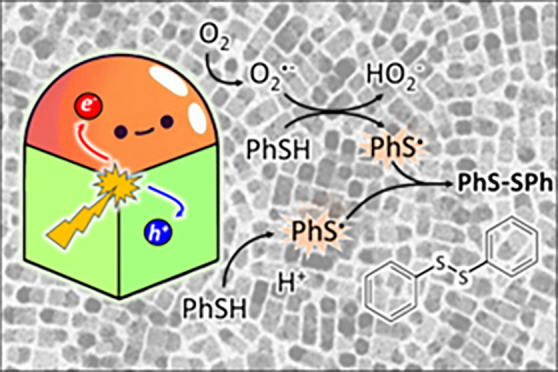

Colloidal nanocrystal heterostructures (HSs) are nanoparticles composed of at least two distinct materials sharing an interface^1,2^ exhibiting properties that can be distinct from their individual components. Semiconductor–semiconductor HSs are especially promising for light-harvesting applications,^3^ as both domains can absorb light and generate electron–hole pairs. Proper band alignment at the heterojunction can enhance charge separation and reduce recombination,^4^ making these architectures attractive for photocatalysis.^5^

HSs based on lead halide perovskites have emerged as promising photocatalysts,^6^ as they combine tunable band gaps^7^ and the intrinsic defect tolerance^8^ of CsPbX_3_ with the additional electronic tunability of heterojunctions. Indeed, significant progress has been achieved using CsPbBr_3_ nanocrystals as photocatalysts for organic transformations.^9^ However, improving the photocatalytic performances of metal halide perovskites, beyond their intrinsic instability under thermal and humid conditions,^10^ requires addressing several crucial factors such as their optical absorption properties, the generation and separation of photogenerated charge carriers, and the surface reaction kinetics. A highly promising strategy to enhance charge separation efficiency is coupling perovskites with a secondary semiconductor. This combination allows for an improved surface reaction rate through advanced configurations, such as type-II, Z-scheme, and S-scheme heterojunctions.^11^ In this context, CsPbX_3_/Pb_4_S_3_Y_2_ (X, Y = Cl, Br, I) perovskite/chalcohalide epitaxial HSs, initially reported by some of the authors,^12^ have recently emerged as promising photocatalysts, where strong interfacial coupling enhances charge separation.^12^ For instance, Pradhan et al.^13^ recently demonstrated that CsPbBr_3_/Pb_4_S_3_Br_2_ HSs outperform CsPbBr_3_ nanocrystals (NCs) in CO_2_ photoreduction due to suppressed radiative recombination and efficient charge transfer across the heterojunction.

Most perovskite-based heterostructure photocatalysts reported in the literature present a nonepitaxial interface between the two semiconductors,^14^ where the benefits of the architecture mainly rely on the heterojunction type rather than on interface quality.^15^ In contrast, the proposed perovskite/chalcohalide CsPbX_3_/Pb_4_S_3_Y_2_ HSs present several advantages over the reported HSs: (a) an epitaxial interface, which enables a potentially defect-free junction, thereby reducing the probability of recombination centers; (b) a type-II heterojunction, with efficient electron delocalization across the nearly isoenergetic conduction bands of both semiconductors; and (c) a dimer morphology that could improve the number of effective contact points with the substrate.

Despite this potential, their application in organic transformations in nonpolar media remains largely unexplored. To address this gap, we investigate the band alignment of three different CsPbX_3_/Pb_4_S_3_Y_2_ (X, Y = Cl, Br, I) HSs, as interfacial energy level matching is crucial to designing an effective photocatalyst for specific target reactions.

Based on this knowledge, we selected the oxidative coupling of thiophenols into disulfides as a proof-of-concept reaction to directly assess how the use of a semiconductor–semiconductor heterostructure catalyst enhances the photocatalytic performance. This model transformation, relevant across (bio)­chemistry^16^ and industry,^17^ enables direct evaluation of the advantages of semiconductor heterojunctions over single-phase nanocrystals. Compared to conventional methods, this photocatalytic approach offers a milder and more sustainable alternative, avoiding overoxidation and simplifying purification.^18^ To assess the photocatalytic activity of the CsPbX_3_/Pb_4_S_3_Y_2_ HSs in the oxidation of *p*-thiophenols, we compared their reactivity against their individual components (CsPbX_3_ and Pb_4_S_3_Y_2_ NCs). Among all HSs we tested, CsPbBr_3_/Pb_4_S_3_Br_2_ showed the highest yield (81%) and selectivity (87%), outperforming stand-alone NCs and suggesting efficient charge separation at the interface. These results were attained in only 90 min upon 450 nm illumination, a significantly shorter time than that in previous studies with perovskite-based photocatalysts.^19^ These results underscore the pivotal role of heterojunction configuration in enhancing charge separation and photocatalytic performance.

The first synthetic protocol developed for CsPbBr_3_/Pb_4_S_3_Br_3_ HSs relied on perovskite nanoclusters as precursors, which were reacted with a sulfur source to obtain the product.^12^ While producing well-defined heterostructures, this protocol has a low reaction yield, hampering extensive testing. Therefore, we here optimized an alternative published protocol,^13^ using preformed CsPbBr_3_ nanocrystals reacted with lead oleate, 1-dodecanethiol (DDT), and sulfur in 1-octadecene (S-ODE). This approach significantly increases the yield (130 mg/batch), albeit at the cost of a less controlled morphology and a higher fraction of stand-alone CsPbBr_3_ nanocrystals (∼20–25%, quantified by analyzing more than 500 NCs from TEM images). Figure 1a is a scheme of the protocol, where CsPbX_3_ NCs synthesized via a hot-injection method^20^ are immediately reacted, without purification, with the chalcohalide precursors injected at 220 °C. “Free-standing” CsPbX_3_ NCs (used later as a reference) were obtained by interrupting the synthesis after the first step, while Pb_4_S_3_Y_2_ nanocrystals were recovered by selective etching of the perovskite domain in the corresponding HSs using dimethyl sulfoxide (DMSO).^21^ Figure 1b shows the typical optical absorption spectra of the HSs and isolated NCs for the X = Y = Br case, with characteristic absorption features of the perovskite highlighted in green and the broad and featureless absorption of Pb_4_S_3_Br_2_ NCs in red. As expected, the HSs absorption spectrum combines both features of the free-standing NCs. Representative TEM images of the three samples are displayed in Figure 1c–e (see Figures S1–S3 for additional characterization data).

**Figure 1:**
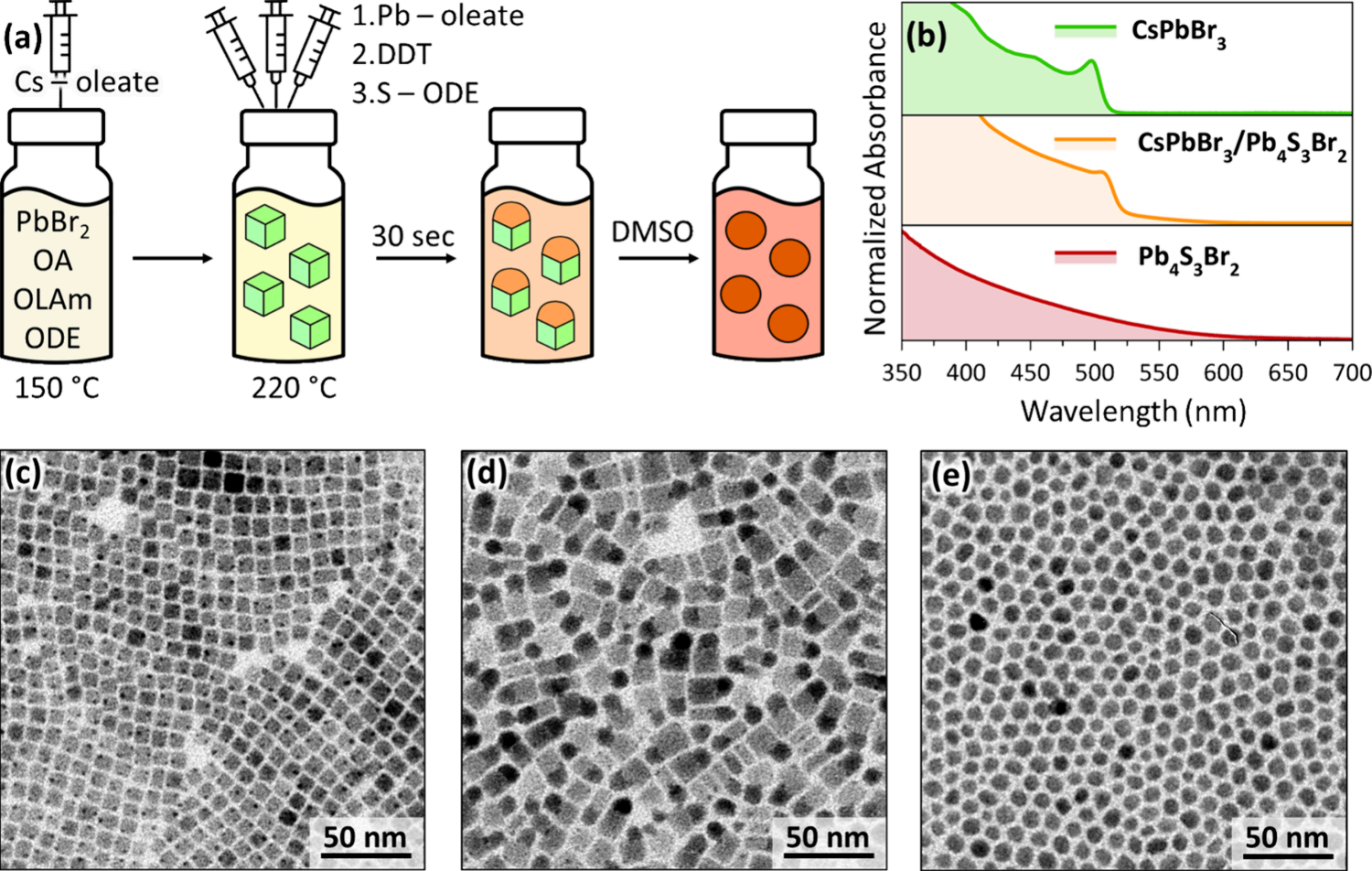
Synthesis of CsPbBr_3_/Pb_4_S_3_Br_2_ HSs. (a) Scheme of the synthetic route used to obtain CsPbBr_3_ NCs and CsPbBr_3_/Pb_4_S_3_Br_2_ HSs, followed by selective etching of the perovskite domain to isolate free-standing Pb_4_S_3_Br_2_ NCs. (b) Absorption spectra of CsPbBr_3_ NCs (green), CsPbBr_3_/Pb_4_S_3_Br_2_ HSs (orange), and Pb_4_S_3_Br_2_ NCs (red). (c–e) TEM images of CsPbBr_3_ NCs (c), CsPbBr_3_/Pb_4_S_3_Br_2_ HSs (d), and Pb_4_S_3_Br_2_ NCs (e).

The presence of two independent semiconductor domains allows for some tuning of the band gap to target specific reactions, in principle expanding the scope of HSs as photocatalysts. For instance, replacing PbBr_2_ with PbCl_2_ in the reaction yields CsPbCl_3_/Pb_4_S_3_Cl_2_ HSs (Figure 2a,b) with only minor adjustments to the synthesis required (see Experimental Methods in the Supporting Information and Figures S4–S7). Conversely, the direct synthesis of CsPbI_3_/Pb_4_S_3_I_2_ HSs was unsuccessful, probably due to the increased lattice mismatch of CsPbI_3_/Pb_4_S_3_I_2_ compared to the Br and Cl analogues.^12^ However, CsPbI_3_-based HSs can still be obtained via halide exchange from CsPbBr_3_/Pb_4_S_3_Br_2_ HSs, as shown by us in a previous work.^12^ Due to the low ionic mobility in the chalcohalide domain, such a reaction primarily affects the perovskite domain,^12^ resulting in CsPbI_3_/Pb_4_S_3_Br_2_ HSs (Figure 2c,d and Figure S8). The exchange was tracked by the redshift of the perovskite absorption onset, while the contribution from the chalcohalides domain remained essentially unchanged (Figure 2c).

**Figure 2:**
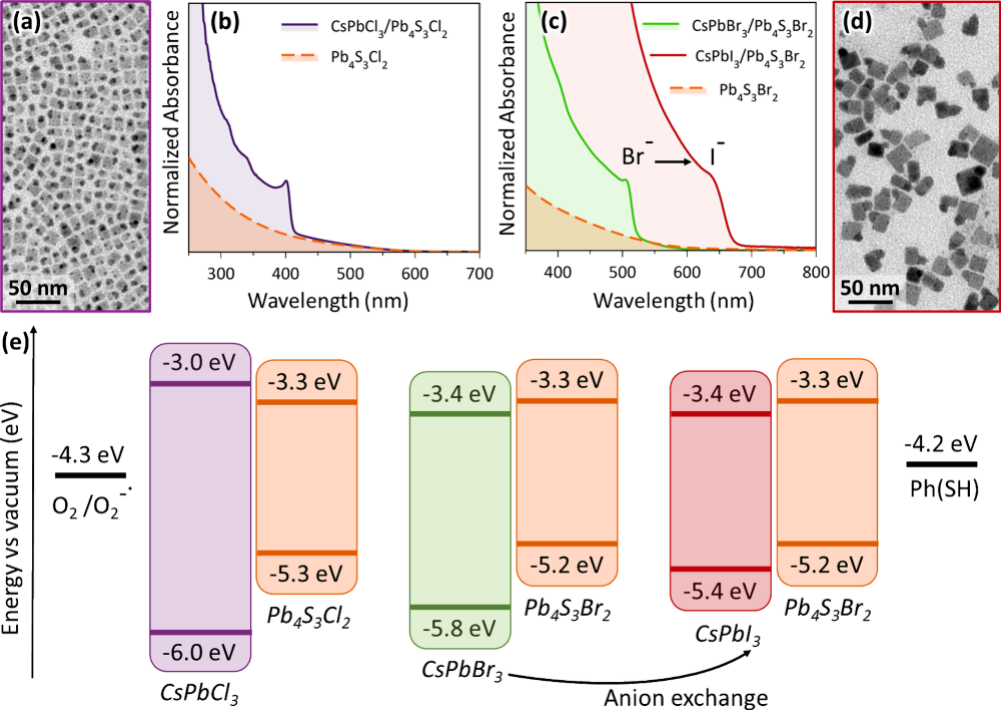
Synthesis of HSs and band alignments. (a) TEM image of CsPbCl_3_/Pb_4_S_3_Cl_2_ HSs. (b) Absorption spectra of CsPbCl_3_/Pb_4_S_3_Cl_2_ HSs (purple line) and Pb_4_S_3_Cl_2_ chalcohalides NCs (orange line). (c) Absorption spectra of CsPbBr_3_/Pb_4_S_3_Br_2_ HSs (green line), CsPbI_3_/Pb_4_S_3_Br_2_ HSs obtained from them by halide exchange (red line), and Pb_4_S_3_Br_2_ chalcohalides NCs (orange line). (d) TEM image of CsPbI_3_/Pb_4_S_3_Br_2_ HSs. (e) Band alignments of the valence band (VB) and conduction band (CB) of different HSs, compared with the highest occupied molecular orbital (HOMO) of thiophenol (PhSH) and the reduction potential of O_2_ to superoxide anion.

The combination of these strategies enables the various CsPbX_3_/Pb_4_S_3_Y_2_ HSs to cover the whole visible spectrum (Figure 2e). The band alignment and redox properties of “free-standing” CsPbX_3_ and Pb_4_S_3_Y_2_ NCs were characterized by combining optical absorption spectroscopy, to determine the optical band gap, with ultraviolet photoelectron spectroscopy (UPS) and ambient pressure photoemission spectroscopy (APS), to establish the position of the valence band (VB) edge with respect to the vacuum level (Figures S9–S13). While the literature is rich in reports on the band positions of CsPbX_3_,^22^ very limited experimental data are available for Pb_4_S_3_Y_2_ chalcohalides.^21,23^ Our results confirm the current computational predictions,^23,12^ highlighting that the band gap of lead chalcohalides is only minimally affected by the halide composition, likely due to the band edge states being predominantly derived from Pb^2+^ (conduction band) and S^2–^ (valence band) orbitals.^23^ We also confirmed the type-I and type-II band alignments predicted respectively for CsPbCl_3_/Pb_4_S_3_Cl_2_ and CsPbBr_3_/Pb_4_S_3_Br_2_ HSs, which explains the photoluminescence quenching observed in both systems. Notably, for the CsPbBr_3_/Pb_4_S_3_Br_2_ HSs the conduction band edges of the two materials are closely aligned in energy (−3.4 eV for CsPbBr_3_ and −3.3 eV for Pb_4_S_3_Br_2_), thus facilitating electron delocalization across the HSs.

According to the energy levels estimated for CsPbX_3_/Pb_4_S_3_Y_2_ and the HOMO value for thiophenol (−4.2 eV, calculated from cyclic voltammetry, Figure S14), the CsPbBr_3_/Pb_4_S_3_Br_2_ HSs present optimal band levels to perform the photocatalytic reaction. In the case of CsPbCl_3_/Pb_4_S_3_Cl_2_ HSs, the type-I alignment would lead to a competitive nonradiative recombination in the chalcohalide domain, limiting charge separation, while CsPbI_3_/Pb_4_S_3_Br_2_ HSs require an additional halide exchange step in the synthesis without offering a sensible advantage over CsPbBr_3_/Pb_4_S_3_Br_2_ HSs.

Based on these considerations, we selected as a benchmark the photooxidation of PhSH (**1a**) to disulfide (**1b**) catalyzed by CsPbBr_3_/Pb_4_S_3_Br_2_ HSs under 450 nm LED excitation (Scheme 1) without an external sacrificial electron donor, as discussed in detail later (see Experimental Methods, Figures S15 and S16, and Table S1).

**Scheme 1:**
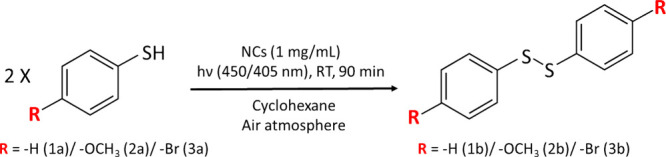
Standard Conditions for the Photooxidative Coupling of *p*-Substituted Thiophenols

Preliminary solvent screening showed that cyclohexane gave the highest yield (81%, Table 1 and Figure S17), outperforming hexane likely due to structural features that enhance the solvation of both nanoparticles and substrate, improving photocatalyst–substrate interactions.^24^ A 1:1 CH_2_Cl_2_/cyclohexane mixture gave a high yield (76%) but lower selectivity, which is unfavorable for further photocatalytic investigations. In contrast, the photoreaction carried out in CH_2_Cl_2_ yielded an even lower yield (33%). This was attributed to the partial anion exchange between Br^–^ in the perovskite domain and Cl^–^ ions from CH_2_Cl_2_ as a result of the C–Cl bond cleavage, as previously reported by Wu et al. (Figure S18).^19^ These results highlight that reaction kinetics depend also on substrate diffusion and solvation energy, with the solvent influencing substrate adsorption on the NCs surface and consequently the S–S bond formation.

**Table 1:** Standard Conditions^a^ Using Different Solvents for the Photooxidative Coupling of Thiophenol

Entry	Solvent	Conversion (%)	**1b** Yield (%)	Selectivity (%)
1	Cyclohexane	93.0	81	87
2	Cyclohexane/CH_2_Cl_2_ (1:1)	98.0	76	76
3	Hexane	88.0	57	65
4	CH_2_Cl_2_	100.0	33	33

a Conditions: thiophenol (0.034 mmol), CsPbBr_3_/Pb_4_S_3_Br_2_ HSs (1 mg) in 1 mL of solvent after 90 min of irradiation (λ_max_ = 450 nm) at 20 °C in air. Yields are determined by GC-MS using biphenyl as the internal standard.

The photocatalytic activity of the HSs was compared with single-component CsPbBr_3_ and Pb_4_S_3_Br_2_ NCs, using 1 mg of photoactive material (estimated by TGA analysis, Figure S19). Although HSs and single-component NCs photocatalyzed the reaction (Table 2), both the product yield and the selectivity were significantly improved for CsPbBr_3_/Pb_4_S_3_Br_2_ HSs; this can be ascribed to the effective charge separation induced by the electronic junction in the HSs. Control experiments conducted in the absence of the photocatalyst or light resulted in only 2% or 10% **1b** yields, respectively, further confirming the photocatalytic activity of the HSs. The turnover number (TON) and turnover frequency (TOF) were determined according to the method previously reported by some of the authors.^25^ The HSs exhibited the highest photocatalytic performance, with a TON of 14 300 and a TOF of 9560, while significantly lower values were obtained for the CsPbBr_3_ (TON = 3680; TOF = 2460) and Pb_4_S_3_Br_2_ nanocrystals (TON = 2070; TOF = 1380). Additionally, the CsPbBr_3_/Pb_4_S_3_Br_2_ HSs outperform other photocatalysts (Table S2) in terms of reduced reaction time with high TON and TOF numbers.

**Table 2:** Standard Conditions Using Different Photocatalysts (Bromide-Based) for the Coupling of Thiophenol and *p*-Substituted Thiophenols

Entry	Variance	R Substituent	Photocatalyst	Conversion (%)	Product Yield (%)	Selectivity (%)
1	None	–H	CsPbBr_3_/Pb_4_S_3_Br_2_	93.0	81 ± 1^a^	87
2	None	–H	CsPbBr_3_	73.4	50 ± 4^a^	72
3	None	–H	Pb_4_S_3_Br_2_	71.6	59 ± 4^a^	86
4	Without photocatalyst	–H	-	19	2	-
5	Without light	–H	CsPbBr_3_/Pb_4_S_3_Br_2_	51	10	-
6	Without light	–H	CsPbBr_3_	45	4	-
7	Without light	–H	Pb_4_S_3_Br_2_	40	7	-
8	In nitrogen	–H	CsPbBr_3_/Pb_4_S_3_Br_2_	61	22	-
9	In nitrogen	–H	CsPbBr_3_	40	2	-
10	In nitrogen	–H	Pb_4_S_3_Br_2_	40	5	-
11	None	–OCH_3_	CsPbBr_3_/Pb_4_S_3_Br_2_	100	94	94
12	None	–OCH_3_	CsPbBr_3_	100	86	86
13	None	–OCH_3_	Pb_4_S_3_Br_2_	100	93	93
14	None	–Br	CsPbBr_3_/Pb_4_S_3_Br_2_	92	61	66
15	None	–Br	CsPbBr_3_	99	53	53
16	None	–Br	Pb_4_S_3_Br_2_	93	44	47

a Obtained from three independent measurements.

To investigate the influence of the *p*-substituent electronic properties on thiophenol reactivity, we selected two representative substrates: 4-bromothiophenol and 4-methoxythiophenol, bearing either an electron-withdrawing (−Br) or electron-donating (−OCH_3_) group. As shown in Table 2, HSs again gave higher product yields than the single-component NCs. Notably, with 4-methoxythiophenol the yield of **2b** was ca. 94% (Figure S20, Table 2, entry 11). This result is consistent with the electron-donating nature of the −OCH_3_ group, which increases the electron density of the aromatic ring, thereby enhancing its reactivity. Conversely, the −Br substituent, being more electron-withdrawing, makes the aromatic ring more electron-deficient, resulting in a lower reactivity (Figure S21, Table 2, entry 14). Electron-donating groups stabilize the thiyl radical by reducing the spin density at the sulfur atom, explaining the highest yield with *p*-OCH_3_ thiophenol.^26^ Figure 3a shows a clear correlation between the sigma value of the substituent (σ_p_)^27^ and the product yield for CsPbBr_3_/Pb_4_S_3_Br_2_ HSs, consistent with the electronic effects.

**Figure 3:**
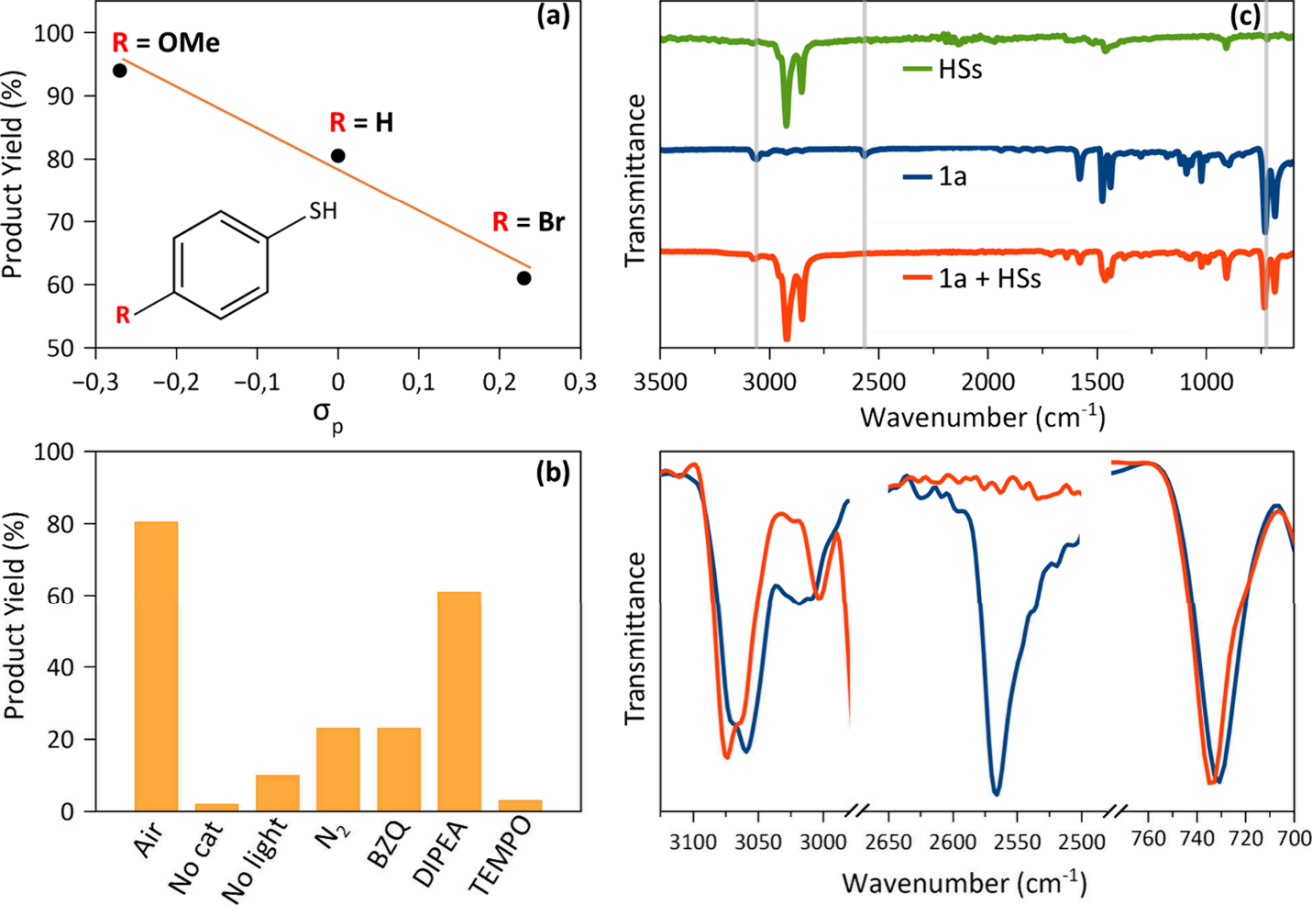
Evaluation of different *p*-substituted thiophenols, different reaction conditions tested, and photocatalyst–substrate interaction. (a) Correlation between the electronegativity of the R substituent on thiophenol and the disulfide yield. (b) Product yield obtained from photocatalytic reactions tested under different conditions and in the presence of radical anions, holes, and radical scavengers. (c) Top: FTIR spectra of CsPbBr_3_/Pb_4_S_3_Br_2_ HSs (green trace), **1a** (thiophenol, blue trace), and a mixture of CsPbBr_3_/Pb_4_S_3_Br_2_ HSs and **1a** (red trace). Bottom: magnification of selected FTIR stretching regions highlighting differences between thiophenol (blue trace) and the HSs–thiophenol mixture (red trace).

To elucidate the photocatalytic mechanism, several experiments with active trapping species were performed, employing 1,4-benzoquinone (BZQ),^28^ *N*,*N*-diiso­propyl­ethyl­amine (DIPEA),^29^ and 2,2,6,6-tetra­methyl­piper­idine-1-oxyl (TEMPO), as shown in Figure 3b and summarized in Table S3. In the presence of BZQ, a well-known scavenger of superoxide anions (O_2_^•–^), **1b** was obtained in 23% yield, which closely matches the yield observed under anaerobic conditions. Conversely, DIPEA, which is a hole (h^+^) scavenger, led to a moderate reduction in yield (61%), suggesting that photogenerated holes also participate, albeit to a lesser extent, in the mechanism (as discussed below). When the reaction was performed in the presence of TEMPO as a radical scavenger, under both aerobic and anaerobic conditions, only negligible amounts of product were obtained (Figure 3b), suggesting the formation of thiyl radicals ( PhS•). The detection of the TEMPO-PhS adduct by GC-MS analysis (Figure S22) confirmed the production of thiyl radicals as key intermediates in disulfide formation.

The surface chemistry of the photocatalyst also plays an important role in the overall photocatalytic activity. The possible thiophenol adsorption onto the NCs surface was investigated by Fourier transform infrared spectroscopy (FTIR), and the spectrum of the CsPbBr_3_/Pb_4_S_3_Br_2_–PhSH mixture was compared with those of the heterostructures and thiophenol alone (Figure 3c, top). In the CsPbBr_3_/Pb_4_S_3_Br_2_–PhSH mixture spectrum, the S–H band at 2565 cm^–1^ is absent, indicating interaction between thiophenol and the HSs. The C–S stretching band shifts from 730 to 735 cm^–1^, and the C–H stretching peak at 3050 cm^–1^ shifts to higher energy (Δν = 14 cm^–1^), reflecting changes in the chemical environment (Figure 3c, bottom). Similar shifts were observed for mixtures of PhSH and “free-standing” NCs, as well as in the case of *p*-OCH_3_ and *p*-Br thiophenol (Figures S23–S26), in accordance with previous reports on organic molecule–perovskite interactions.^30^ Collectively, these data support the adsorption of the different substrates onto the surface of the photocatalyst, which likely facilitates photogenerated charge transfer from the HSs to the substrate.

The proposed mechanism for the photooxidation of *p*-substituted thiophenols under aerobic conditions is represented in Figure 4. Once the contact between the photocatalyst and the substrate is established, upon visible-light illumination, the photogenerated holes migrate toward the Pb_4_S_3_Br_2_ domain, while electrons are delocalized across the conduction band of the heterostructure. This partial charge separation can reduce charge recombination and thus enhance photocatalytic activity compared to the individual components. Under aerobic conditions, two parallel pathways might produce thiyl radicals: (i) photogenerated electrons from the conduction band of the heterostructure reduce O_2_ to form a superoxide anion (O_2_^•–^), which reacts with thiophenol, producing a thiyl radical (PhS^•^) and HO_2_^–^; and (ii) photogenerated holes react with thiophenol, also forming a thiyl radical (PhS^•^) and a proton (H^+^). Subsequently, two thiyl radicals can couple to form the target disulfide product. As the intermediate HO_2_^–^ is unstable, it can react with H^+^ to produce H_2_O_2_. These finding confirm the cooperative activity of both photogenerated charge carriers (electrons and holes) under aerobic conditions to produce thiyl radicals, thus improving the photocatalytic activity in the heterostructure. In a nitrogen atmosphere, there is only one pathway to produce thiyl radicals: the photogenerated electrons and holes interact with the thiophenol, leading to the formation of thiyl and hydrogen radicals, which after coupling produce the disulfide compound and molecular hydrogen (H_2_, identified by GC, Figure S27), respectively (Figure S28). Under both aerobic and anaerobic conditions, the coupling of two thiyl radicals leads to the formation of a disulfide product, which subsequently detaches from the photocatalyst surface due to a lower affinity and diffuses into the solvent.

**Figure 4:**
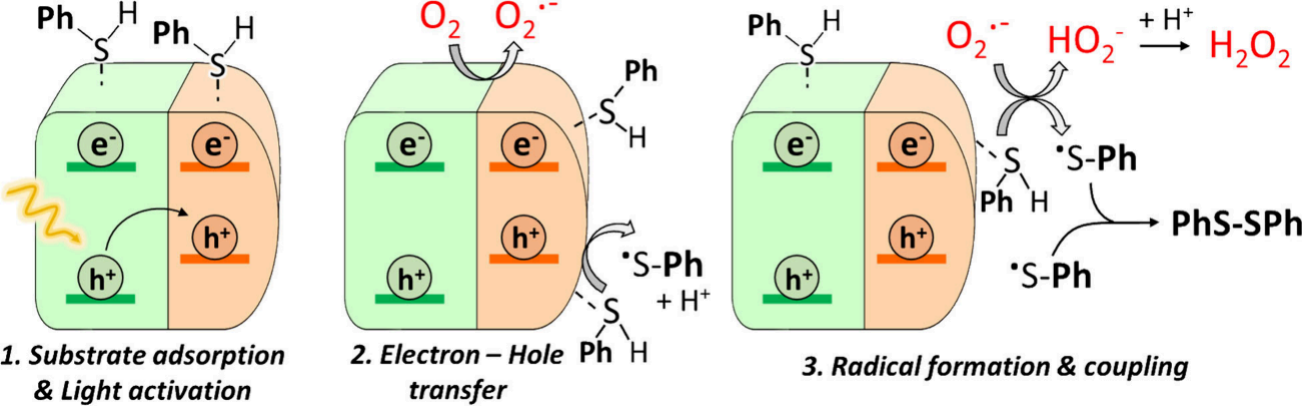
Proposed photocatalytic mechanisms for the oxidative coupling of thiophenol under aerobic conditions.

The performance of the photocatalyst was evaluated after one photocatalytic cycle. XRD patterns recorded before and after the reaction confirmed the preservation of crystallinity of the HSs (Figure S29). The absorption spectrum of CsPbBr_3_/Pb_4_S_3_Br_2_ HSs retained the perovskite band edge with a slight redshift and increased scattering (Figure S30), likely due to some nanocrystal aggregation caused by the partial removal of surface ligands during the photoreaction, as previously observed in C–C coupling with CsPbBr_3_ NCs.^25^ TEM imaging of the crude solution (Figure S31) revealed significant aggregation in part of the sample, but the heterostructure composition was preserved.

To further validate our initial selection criteria for CsPbBr_3_/Pb_4_S_3_Br_2_ HSs, the photoreactions were performed also using Cl HSs (Table S4) and mixed I/Br HSs (Table S5). Employing CsPbCl_3_/Pb_4_S_3_Cl_2_ HSs, the observed trend is consistent with that found for the bromide analogue: the HSs outperform the individual NCs in both yield and selectivity. The slightly lower yield (70% versus 80%) obtained with the Cl HSs can be attributed to the type-I band alignment, which promotes nonradiative recombination and limits charge separation. The CsPbI_3_/Pb_4_S_3_Br_2_ HSs, obtained via anion exchange, delivered the coupling product in 80% yield for **1b**, similar to that obtained with the bromide-based system, consistent with the band alignment. Nonetheless, in all cases, a reduction in the selectivity was observed.

In conclusion, we developed an optimized direct synthesis for semiconductor–semiconductor HSs (CsPbBr_3_/Pb_4_S_3_Br_2_, CsPbCl_3_/Pb_4_S_3_Cl_2_) and CsPbl_3_/Pb_4_S_3_Br_2_ via postsynthetic anion exchange, achieving tens-of-milligrams yields, above those of typical literature reports, and enabling extensive use in organic transformation photocatalysis. The chemical tunability of the perovskite allowed modulation of the band alignment, systematically studied through optical and spectroscopic methods. Using *p*-substituted thiophenol coupling as a model reaction, the HSs outperformed the “free-standing” nanocrystals. Under aerobic conditions, the photogenerated electrons and holes produced thiyl radicals, enhancing the photocatalytic efficiency and exploiting the charge carrier separation at the heterojunction. This work establishes a versatile strategy for constructing semiconductor–semiconductor heterostructures. It demonstrates that the combination of type-II heterojunction engineering, surface chemistry, and the cooperative participation of photogenerated charge carriers to produce intermediate radicals is an effective approach for enhancing the photocatalytic performance of perovskite NCs.

## Supplementary Material



## Notes

Experimental methods; characterization of the photocatalysts by TEM images, ABS/PL spectra, XRD patterns, UPS spectra, XPS spectra, Tauc plots, and TGA curves; product identification by GC-MS; FTIR spectra of different substrates and photocatalysts; and tables of different photocatalysts tested (PDF)
