# Supplementary material for: Metal Halide Perovskite/Chalcohalide Heterojunctions for the Photoinduced Oxidative Coupling of p‑Substituted Thiophenols
Source: ACS Appl Nano Mater. 2026 Mar 6;9(11):4841–7. doi: 10.1021/acsanm.5c05834 (PMC13010340; doi:10.1021/acsanm.5c05834)
Supplement: Supplementary file 1 [file an5c05834_si_001.pdf]

SUPPORTING INFORMATION

Metal Halide Perovskite/Chalcohalide Heterojunctions for the Photoinduced Oxidative Coupling of *p*-Substituted Thiophenols

Anna Cabona,[1,2,4] Stefano Toso,[1,3] Alejandro Cortés-Villena,[4] Ignacio Rosa-Pardo,[4] Mirko Prato,[5] Michele Ferri,[1] Julia Perez-Prieto,[4] Ilka Kriegel,[2]* Liberato Manna[1]* and Raquel E. Galian[4]**

[1] Nanochemistry Department, Italian Institute of Technology, 16163 Genova, Italy;

[2] Department of Applied Science and Technology, Politecnico di Torino, 10129 Turin, Italy;

[3] Lund University, Division of Chemical Physics, Naturvetarvägen 14, 221 00 Lund, Sweden;

[4] Institute of Molecular Science, University of Valencia, c/Catedrático José Beltrán Martínez 2, 46980 Paterna, Valencia, Spain;

[5] Materials Characterization, Italian Institute of Technology, 16163 Genova, Italy;

Corresponding Authors

Julia Pérez-Prieto: julia.perez@uv.es, Ilka Kriegel: ilka.kriegel@polito.it, Liberato Manna: liberato.manna@iit.it, Raquel E. Galian: raquel.galian@uv.es

EXPERIMENTAL METHODS

Chemicals. 1-Octadecene (ODE, tech, 90%), oleic acid (OA, tech, 90%), oleylamine (OLA, tech, 70%), lead(II) bromide ($PbBr_2$, 98%), lead(II) chloride ($PbCl_2$, 98%), lead(II) iodide (PbI_2, 98%), cesium carbonate (Cs_2CO_3, 99%), 1-dodecanethiol (DDT, 99.9%), sulfur powder (S, 99.99%), lead acetate trihydrate ($Pb(OAc)_2{\cdot}3H_2O$, 99.99%), dimethyl sulfoxide (DMSO, 99.5%), were purchased from Sigma-Aldrich. All reagents were used as received without any further experimental purification.

Preparation of Cs-oleate Stock Solution. In a typical synthesis, 120 mg (0,37 mmol) of Cs_2CO_3 were mixed with 1.75 mL of OA and 15 mL ODE in 50 mL three-neck flask, dried for 1h at 110°C and then heated under N_2 to 150°C until the solution turned clear. Thereafter, the solution was transferred into N_2-filled glass vials. The solution is solid at room temperature and needs to be pre-heated before using it.

Preparation of $Pb(OA)_2$ Stock Solution. $Pb(OAc)_2{\cdot}3H_2O$ powder (0.38 g, 1 mmol) and OA (950 uL) were mixed with ODE (9.35 mL) in a 50 mL three-neck round-bottom flask. The reaction mixture was degassed under vacuum for 1 h at 110°C and then heated under N_2 to 150°C until all $Pb(OAc)_2{\cdot}3H_2O$ reacted with OA. Thereafter, the solution was cooled to room temperature (25 °C) and transferred into N_2-filled glass vials.

Preparation of S-ODE Stock Solution. 1 mmol of S powder was mixed with 10 mL ODE (predegassed at 120°C for an hour) in a 20 mL glass. Then, the resulting mixture was sonicated until the complete dissolution of S (ca. 20 min).

Synthesis of $CsPbBr_3/Pb_4S_3Br_2$ HSs. A typical HS synthesis involves two steps, performed consecutively: synthesis of $CsPbBr_3$ nanocrystals and growth of $CsPbBr_3/Pb_4S_3Br_3$ HSs. In the first step, 72 mg of $PbBr_2$ are mixed with 5 ml of octadecene, 50 μL of oleic acid, and 500 μL of oleylamine inside a 20 mL glass vial, under nitrogen atmosphere. The mixture is heated to 170°C to achieve the full solubilization of the powder, followed by the injection at 150°C of 500 μL of a Cs-oleate solution

is injected to initiate the growth of perovskite nanocrystals (NCs). After 5 seconds, the reaction is quenched by immersion in a water bath. The reaction batch is then stirred for 5 minutes at room temperature without performing any purification. Subsequently, the vial is reheated to 220°C, and the precursors needed for the growth of the chalcohalide are injected: 800 μL of lead oleate, 32 μL of 1-dodecanethiol and 480 μL of sulfur-ODE. The reaction is allowed to proceed for 30 seconds before being quenched in an ice water bath. The crude solution is then centrifuged at 6000 rpm for 5 minutes to isolate the NCs. The precipitate is collected in hexane, followed by a second centrifugation at a 2000 rpm for 2 minutes to remove larger perovskite NCs that may have formed during the re-heating step. The precipitate is discarded, and the supernatant is kept for further use.

Synthesis of $CsPbCl_3/Pb_4S_3Cl_2$ HSs. The synthesis of Cl-based HSs follows the same protocol adopted for Br-based HSs, with the following adaptations: 1) 53 mg of $PbCl_2$ were used instead of $PbBr_2$ and the amount of oleic acid was increased to 500 μL to facilitate the dissolution of $PbCl_2$, 2) the first injection temperature was raised to 185°C, as lower temperature (150°C) resulted in a high number of platelets instead of nanoparticles. The procedure to obtain and purify the HSs is the same as the bromide-based sample.

Isolation of $Pb_4S_3X_2$ nanocrystals. Pure $Pb_4S_3X_2$ nanocrystals can be obtained by etching the perovskite domains of the corresponding $CsPbX_3/Pb_4S_3X_2$ HSs. First, a solution of HSs in hexane is centrifuged at 2000 rpm for 2 minutes to remove aggregates, which ensures uniform and effective etching. Subsequently, 1 mL of the HS solution in hexane is mixed with 1 mL of dimethyl sulfoxide and 60 μL of oleylamine. The mixture is vortexed for approximately 60 seconds. After a few minutes, phase separation occurs due to the mutual insolubility and density difference between hexane and dimethyl sulfoxide. The upper hexane layer, which appears red due to the presence of chalcohalide nanoparticles, is collected. After that, 4 mL of ethyl acetate and 60 μL of oleic acid are added to the hexane fraction to precipitate the particles, which are collected by centrifugation (6000 rpm for 5 minutes) and then redispersed in hexane or toluene for further use.

Anion exchange. The anion exchange reactions were performed under ambient conditions following previously reported methods.[1] First, a PbI_2 stock solution was prepared: 2 mmol of PbI2, 5 mL of OA and 5 mL of OLAm were mixed with 30 mL of ODE in a 100 mL three-neck flask. The reaction mixture was dried/degassed under vacuum for 30 min at 110°C. Then, the flask was filled with N_2, and the temperature was raised to 150°C. After complete dissolution of PbI_2 salt, the solution turned yellow and then cooled down to room temperature (25°C) and transferred into a N_2-filled glass vial. Then, 4 mL of the $CsPbBr_3$–$Pb_4S_3Br_2$ HSs in toluene was added into a 10 mL glass vial, and different amounts of PbI_2 stock solution (ranging from 300 uL to 900 μL) were added under vigorous stirring at RT for at least 30 min. Thereafter, the HSs were collected by centrifugation at 6000 rpm for 5 min and redispersed in toluene for further use.

Optical Properties. Absorbance spectra were measured with a Cary300 UV–Vis absorption spectrophotometer, while PL spectra were recorded by a Varian Cary Eclipse spectrophotometer using an excitation wavelength at 350 nm for the bromide-based samples and 300 for the chloride-based ones. The NC solutions were diluted in hexane in quartz cuvettes (path length = 1 cm) to a maximum optical density below 1.0.

Transmission Electron Microscopy. Bright Field Transmission Electron Microscopy (BF-TEM) measurements of the NCs were performed using a JEOL JEM-1011 with a W thermionic source at an acceleration voltage of 100 kV. The highly diluted NC solution was drop-cast onto copper grids (200 mesh) with carbon film, and the solvent was then allowed to evaporate in a vapor-controlled environment. The longitudinal and lateral dimensions were assessed through statistical analysis of TEM images of several hundred NCs using the ImageJ software.

X-ray Powder Diffraction. Characterization by X-ray Powder Diffraction (XRD) was performed by employing a PANalytical Empyrean X-ray diffractometer using a 1.8 kV Cu Kα ceramic X-ray tube operating at 45 kV and 40 mA and detected by a PIXcel3D 2 × 2 area detector. Samples were prepared by drop-casting highly concentrated solutions on zero-diffraction silicon substrates. All

diffraction patterns were acquired at room temperature under ambient conditions. Data analysis was performed using the HighScore 4.9 software from PANalytical.

X-ray Photoelectron Spectroscopy. XPS specimens were prepared by drop casting a few microliters of the sample dispersions onto freshly cleaved highly oriented pyrolytic graphite (HOPG, ZYB grade) substrates. XPS data were acquired using a Kratos Axis UltraDLD spectrometer, equipped with a monochromatic Al Kα source operated at 20 mA and 15 kV. Wide scans were acquired at a pass energy of 160 eV, energy step of 1 eV, over an analysis area of 300 x 700 µm2. Spectra have been charged corrected, the lowest binding-energy component of the carbon 1s spectrum was set at 284.8 eV. The collected data were then analyzed with CasaXPS software (version 2.3.24).[2]

Ultraviolet Photoelectron Spectroscopy. UPS was carried out on the same spectrometer, using a He I (21.22 eV) discharge lamp, on an area of 55 µm in diameter, at a pass energy of 10 eV and with a dwell time of 100 ms. The work function (that is, the position of the Fermi level with respect to the vacuum level) was measured from the threshold energy for the emission of secondary electrons during He I excitation. A −9.0 V bias was applied to the sample to precisely determine the low-kinetic-energy cutoff, as discussed in ref[3]. The position of the cutoff was then estimated with CasaXPS software, using the "Edge Up" background function for the energy interval around the cutoff. Then, the position of the VBM versus the vacuum level was estimated by measuring its distance from the Fermi level, focusing on the high-kinetic energy (i.e., low-binding energy) cutoff region and using the "Edge Down" background function in CasaXPS software.

Ambient Pressure Photoemission Spectroscopy. The ionization (I) energy or valence band (VB) energy of the studied materials was determined using an Ambient Pressure Photoemission Spectroscopy (APS) system (model APS02, KP Technology Ltd, Highlands and Islands, United Kingdom). The APS02 system was operated with an incident photon energy range from 4.56 to 6.89 eV, generated using a tunable UV light source of Deuterium. The samples for APS measurements were prepared by drop-casting thin films of the materials onto ITO-coated glass substrates, which provide a conductive and transparent support compatible with photoemission studies. The APS

chamber was continuously purged with nitrogen gas during operation to effectively suppress ozone formation throughout the measurement. The onset of the photoemission signal with the background was used to determine the VB energy.

Electrochemistry. Electrochemical characterization was performed on an Autolab 128N potentiostat/galvanostat using a three-electrode system in a glass beaker. Cyclic voltammetry (CV) experiments were carried out in 0.1 M tetrabutylammonium tetrafluoroborate ($TBABF_4$) solution in a mixture of anhydrous ACN:toluene (1:3 v/v) with a thiophenol concentration of 0.12 M. A glassy carbon, Pt wire and Ag/AgCl were used as working, counter and reference electrodes, respectively. The measurements were performed at room temperature (298 ± 1 K) and under aerated conditions, with a scan rate of 100 mV/s. To determine the energy of the highest-occupied molecular orbital (HOMO) level, the anodic peak potential (in volts) was referred to the Fc/Fc+ redox couple using 0.5 mM solutions of ferrocene in 0.1 M $TBABF_4$ in the same electrolyte.

Thermogravimetric analysis. Thermogravimetric analysis (TGA) of the NCs was carried out with a Mettler Toledo TGA/SDTA851e/SF/1100 apparatus in the 25-800°C temperature range under a 10°C min' scan rate under nitrogen atmosphere.

Attenuated total reflectance-Fourier transform infrared spectroscopy. To ascertain the substrate approach to the photocatalysts NCs surface, the NCs and the substrates were mixed at the same concentration used in the photocatalytic reactions and the mixture was stirred to ensure the interaction between them. Then, the ATR-FTIR analyses were performed in a Bruker alpha II spectrometer by drop-casting the colloidal mixtures.

Photocatalytic Experiments. For the photocatalytic experiments, 1 mg of the photocatalyst was mixed with the stock solution of the substrate (thiophenol/ 1,4-bromothiophenol/ 1,4-methoxythiophenol) in 1 mL of solvent (cyclohexane) to reach the final concentration of 34 mM. Subsequently, the photoreactions were performed in a 10 mL gastight crimped vials under stirring (160 rpm) using an orbital shaker in a photoreactor with blue LEDs (450/405 nm) for 90 min at 20°C. After the reaction time, the crude solution was centrifuged at 12500 rpm for 15 minutes, the

precipitate was collected in 1 mL of hexane for further use while the supernatant was analyzed by GC-MS to determine the conversion of the substrate and the yield of product. For the GC analysis the biphenyl (internal standard) stock solution was added to the supernatant to have a final concentration of 150 mM. Biphenyl was solubilized in ethyl acetate.

Control experiments of the photocatalytic coupling of thiophenol. All the reactions have been performed in the same conditions reported for the photocatalytic reactions but in the presence of the scavengers. Regarding the control experiments, 10 equivalents of 1,4-benzoquinone, *N,N*-Diisopropylethylamine (DIPEA) and 2,2′,6,6′-tetramethylpiperidine-1-oxyl (TEMPO) were added as scavengers.

Control experiments in nitrogen atmosphere for the detection of H_2. All the reactions under a nitrogen atmosphere for H_2 detection were performed using strictly anhydrous solvents inside a glovebox to prevent any moisture contamination from the atmosphere.

Photocatalytic Setup. Light source: the reactions were performed using (1= 450‡10 m or 1= 405‡10 m) LUXEON LED, mounted on a 10mm Square Saber - 1030 mW@700mA as a light source. Temperature Control: reaction temperature was controlled by a high-precision thermoregulation Hubber K6 cryostat. Likewise, to guarantee stable irradiation the temperature of the LEDs was set up at 20°C. The reactions have been carried out in an in-house parallel High Throughput Screening (HTS) (see Picture 1) photoreactor with capacity to set up to 25 reactions with different excitation wavelengths, respectively, under high-intensity irradiation. These unique HTS platforms allow for tight control of the light intensity and the temperature of the reactions. The 25-positions photoreactor is operable at 1-15 mL reaction volumes for each reaction.

Gas chromatography – mass spectroscopy. The work-up of the reaction was performed by adding biphenyl (IS) to the crude. Then, 0.1 mL of the mixture was diluted with 0.9 mL of cyclohexane. The solution was injected into the GC-MS (Agilent 7890B - 5977A) and the products were identified according to the retention time and mass. A calibration curve was established using different concentrations of analytes (C_{AN}) relative to a fixed concentration of the internal standard (C_{IS} = 150

mM). The GC signals were then analyzed by comparing the peak areas of the analytes with that of the internal standard. The ratio between the product area and the internal standard allowed us to quantify the product concentrations in the reactions. This calibration plot was subsequently used to quantify the obtained products.

The following equations were used to calculate the product yield (1), substrate conversion (2) and the selectivity (3):

$\boldsymbol{Product\ Yield}$ (%) = $[\boldsymbol{Pro}(\boldsymbol{AN})]/\ [\boldsymbol{Initial\ substrate}]\ \boldsymbol{x100}\%$ (1)

$\boldsymbol{Conversion}$ (%) = $\mathbf{100}-\ [\boldsymbol{Final\ substrate}]/\ [\boldsymbol{Initial\ substrate}]\ \boldsymbol{x100}\%$ (2)

Selectivity (%) = $[\boldsymbol{Product\ Yield}]/[\boldsymbol{Conversion}]\boldsymbol{x100}\%$ (3)

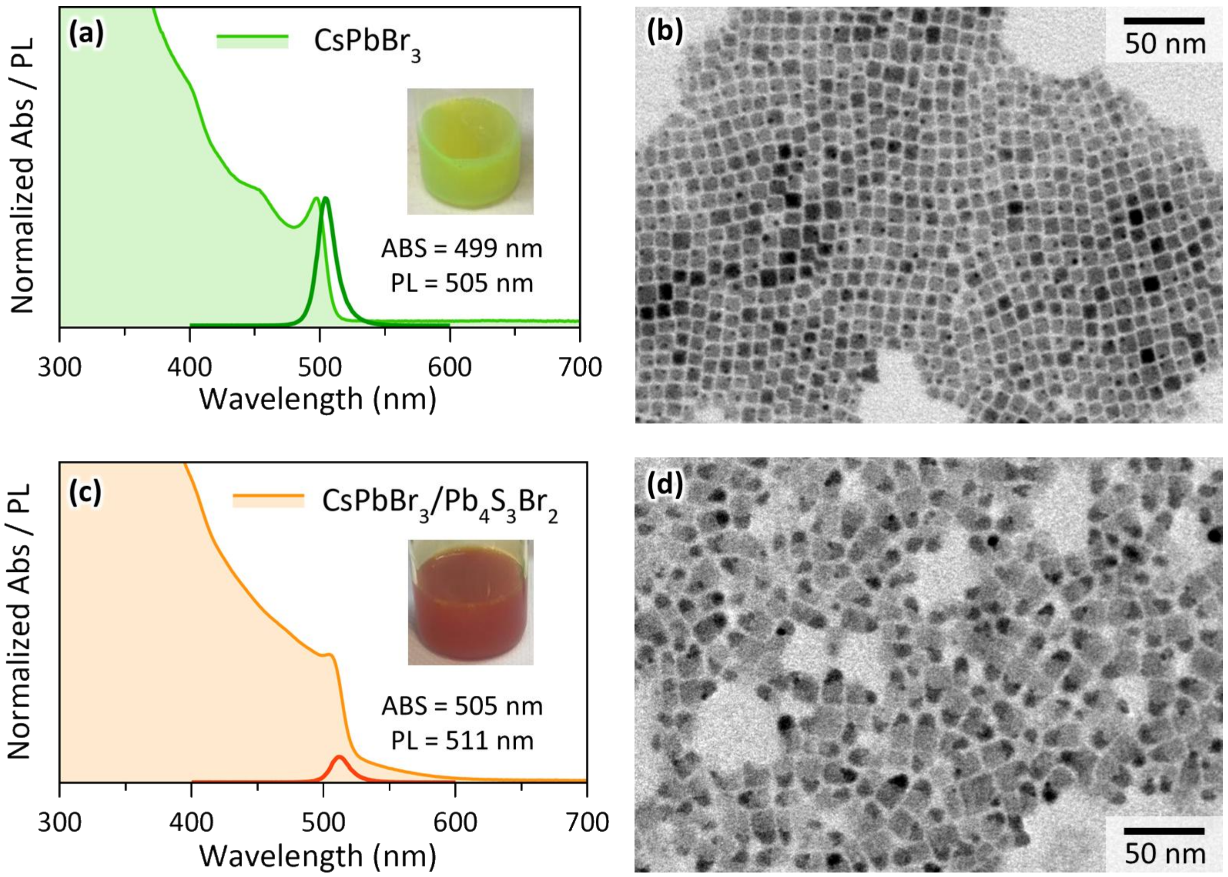

Figure S1. Optical characterization and TEM images of $CsPbBr_3$ and $CsPbBr_3/Pb_4S_3Br_2$ heterostructures. a) Absorption and PL spectra of $CsPbBr_3$ NCs synthetized at 150 °C prior to the growth of the chalcohalide domains. b) TEM images of the same $CsPbBr_3$ nanocrystals. c) Absorption and PL spectra of $CsPbBr_3/Pb_4S_3Br_2$ heterostructures after the growth of the chalcohalide domains. The PL was acquired with the same dilution factor and conditions of panel (a) and shows a marked reduction in intensity compatible with the formation of heterostructures, which are known to suppress the photoluminescence of the $CsPbBr_3$ domain. d) TEM images of $CsPbBr_3/Pb_4S_3Br_2$ heterostructures. Comparing the position of the absorption features, and TEM images before and after the growth of the chalcohalide, highlights some enlargement of the $CsPbBr_3$ domains, whose average size increases from 9.1 nm to 11.7 nm. This is likely due to the reaction of some of the additional Pb-oleate with unreacted Cs^+ in present in the reaction environment.

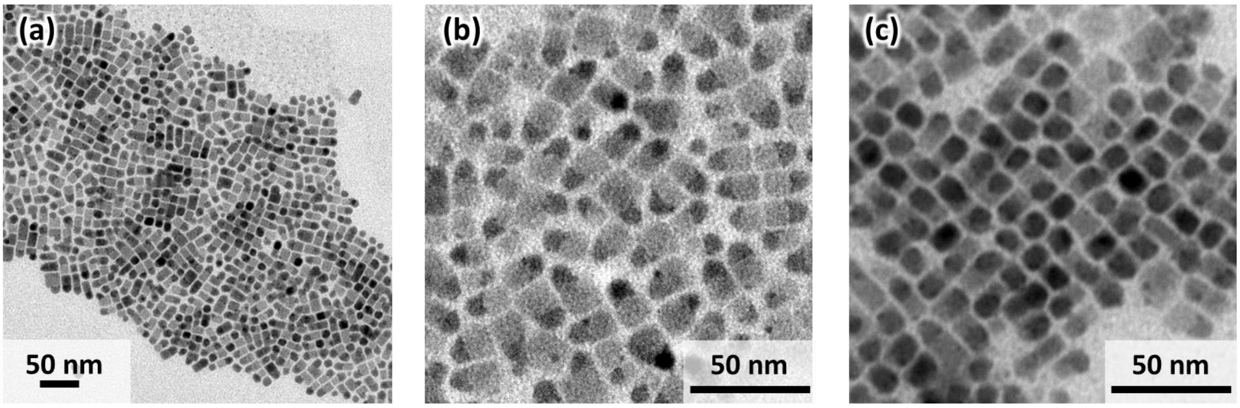

Figure S2. TEM images of $CsPbBr_3/Pb_4S_3Br_2$ heterostructures at different magnifications. The different projections visible in panel (a) allow to see some heterostructures lying flat on the TEM grid (b) (rectangular cross-section) as well as some standing upright (c) (squared cross-section).

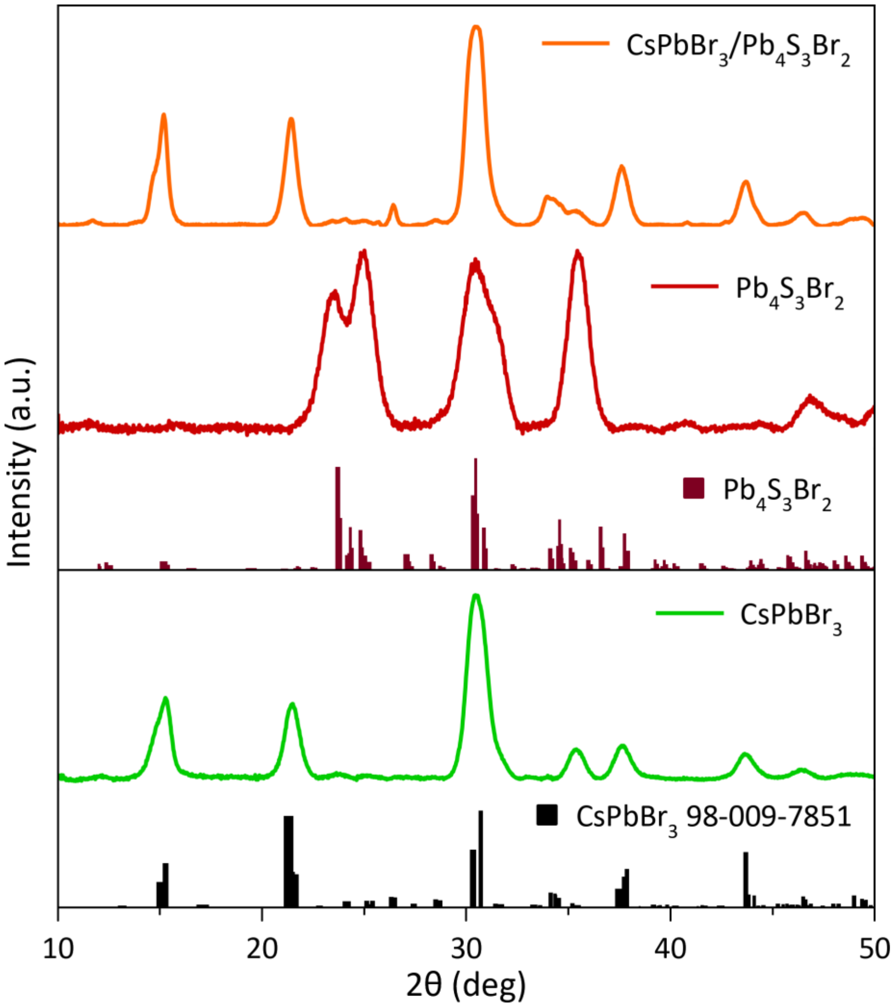

Figure S3. XRD patterns $CsPbBr_3$ and $Pb_4S_3Br_2$ free-standing nanocrystals and heterostructures. XRD pattern of $CsPbBr_3/Pb_4S_3Br_2$ HSs (orange line), $Pb_4S_3Br_2$ NCs (red line) and reference pattern (bordeaux line), $CsPbBr_3$ (green line) and $CsPbBr_3$ reference (black line). The contribution of $Pb_4S_3Br_2$ to the heterostructures pattern is minor due to the much lower volume fraction compared to $CsPbBr_3$ and to the spread of diffracted intensities over more peaks due to the lower symmetry (although both materials crystallize in the Pnma space group, the pseudocubic symmetry of $CsPbBr_3$ causes many broad reflections to stack onto each other).

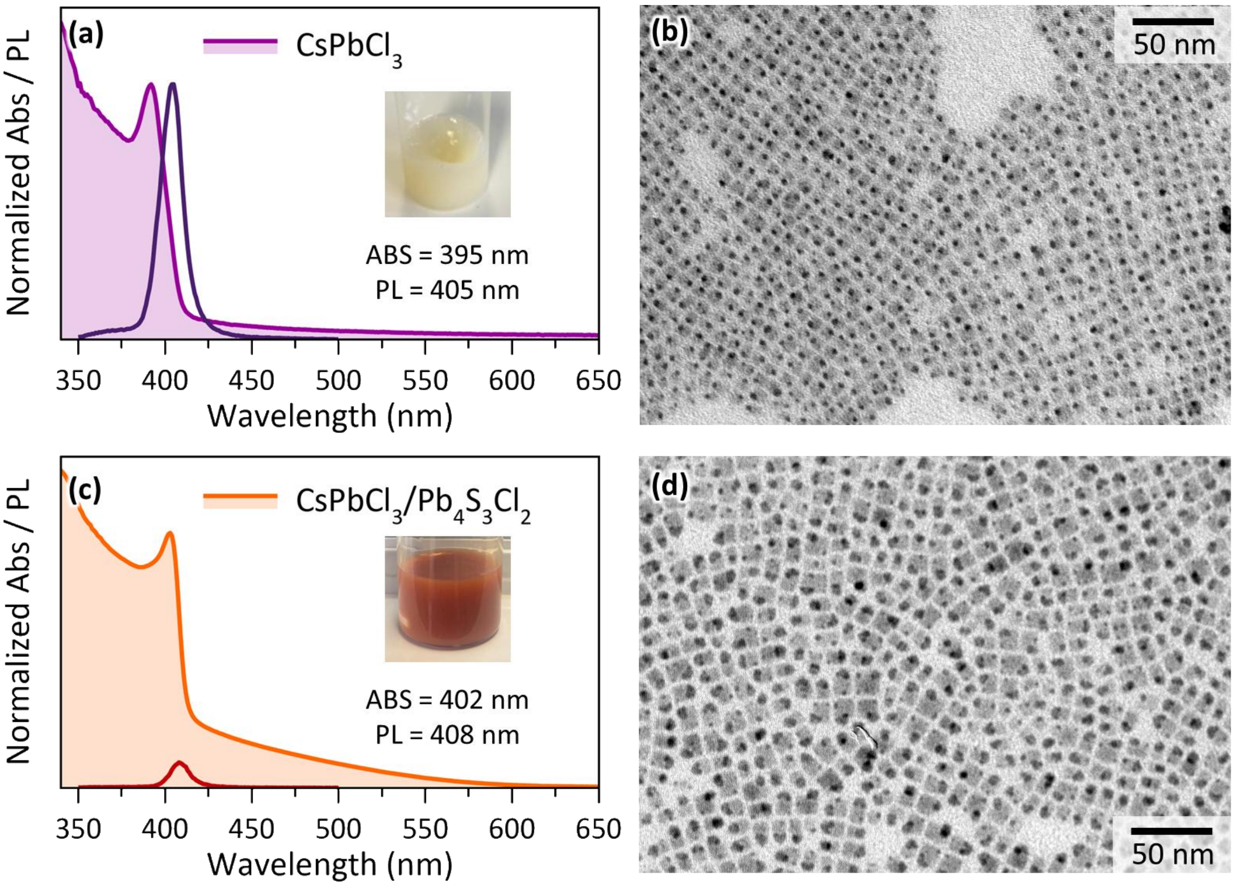

Figure S4. Optical characterization and TEM images of $CsPbCl_3$ nanocrystals and $CsPbCl_3/Pb_4S_3Cl_2$ heterostructures. a) Absorption and photoluminescence spectra of $CsPbCl_3$ nanocrystals synthetized at 185 °C. b) TEM images of the same $CsPbCl_3$ nanocrystals. c) Absorption and photoluminescence spectra of $CsPbCl_3/Pb_4S_3Cl_2$ heterostructures. d) TEM images of the same $CsPbBr_3/Pb_4S_3Cl_2$ heterostructures.

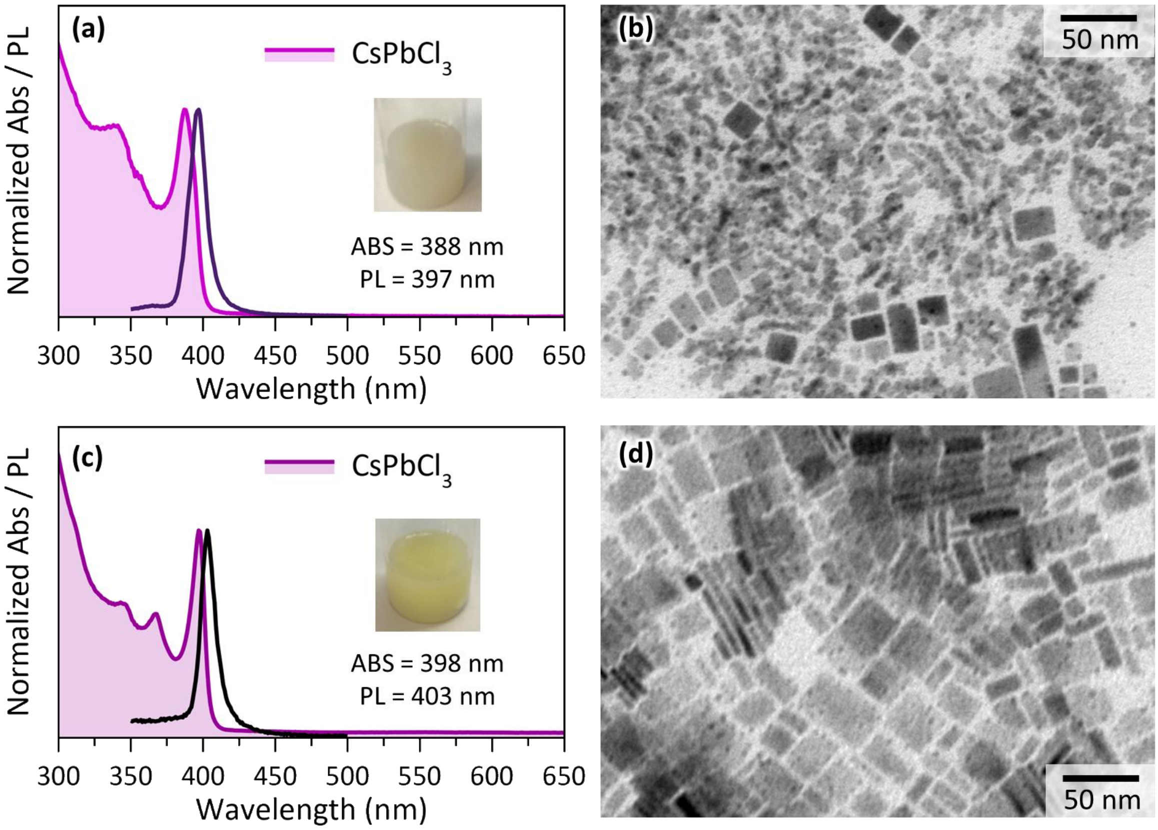

Figure S5. Optimization of the $CsPbCl_3$ nanocrystal seeds for heterostructure growth. a) Absorption and photoluminescence spectra of $CsPbCl_3$ nanocrystals synthetized at 150 °C and with the use of TOP (trioctylphosphine). b) TEM image of the same $CsPbCl_3$ nanocrystals. c) Absorption and photoluminescence spectra of heterostructures growth tests performed on the $CsPbCl_3$ nanocrystals (panel b). d) TEM image of the same test, no heterostructures are found under these conditions.

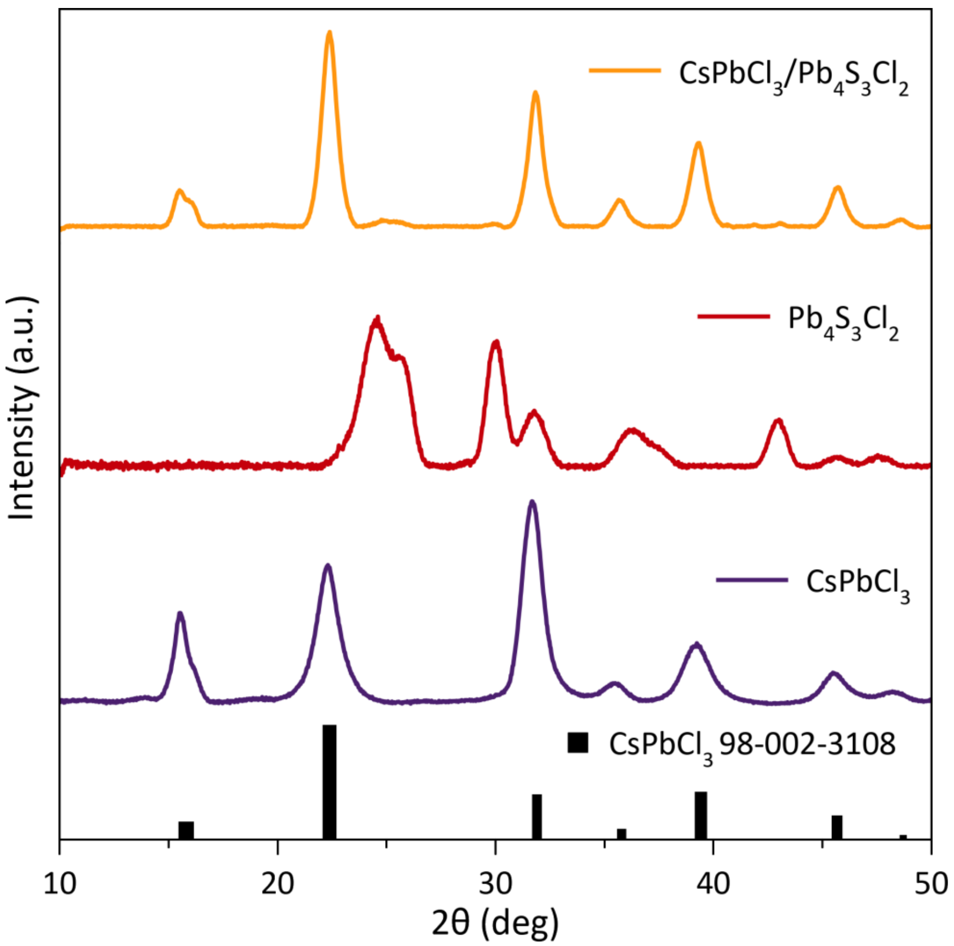

Figure S6. XRD patterns $CsPbCl_3$ and $Pb_4S_3Cl_2$ free-standing nanocrystals and heterostructures. XRD pattern of $CsPbCl_3/Pb_4S_3Cl_2$ HSs (orange line), $Pb_4S_3Cl_2$ NCs (red line) and $CsPbCl_3$ (purple line) $CsPbCl_3$ reference (black line). The contribution of $Pb_4S_3Cl_2$ to the heterostructures pattern is minor due to the much lower volume fraction compared to $CsPbCl_3$ and to the spread of diffracted intensities over more peaks due to the lower symmetry.

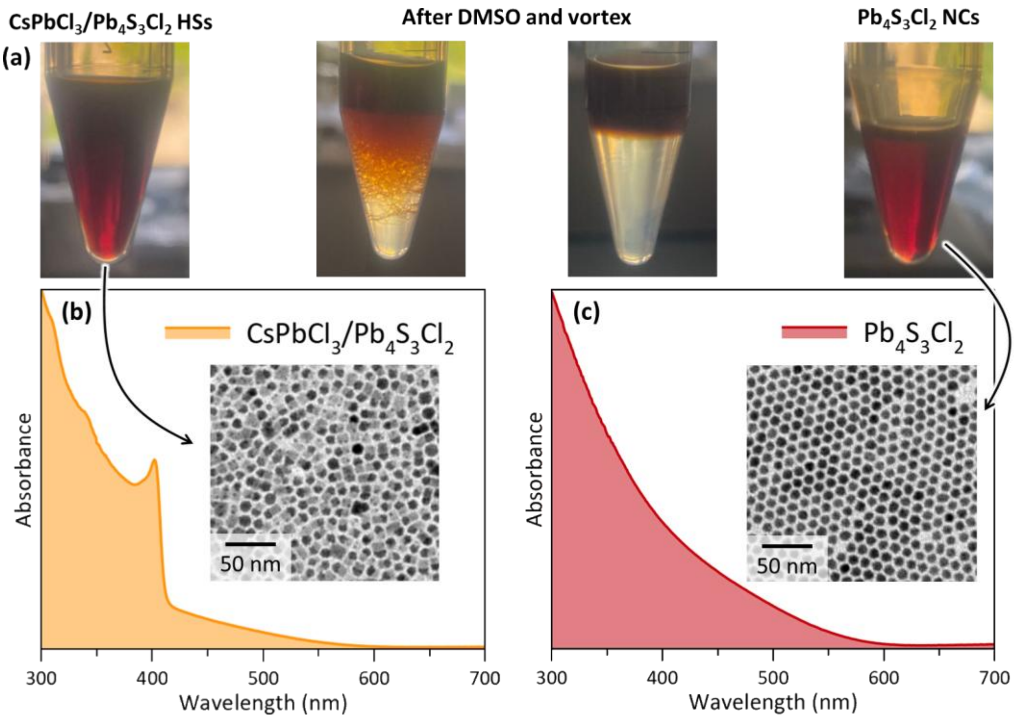

Figure S7. Photographs of $CsPbCl_3/Pb_4S_3Cl_2$ HSs samples before and after selective perovskites etching, and corresponding optical absorbance and TEM images. a) Photographs of heterostructures sample before and after the addition of DMSO to etch the perovskites domain. b) Absorbance spectrum of $CsPbCl_3/Pb_4S_3Cl_2$ HSs before addition of DMSO, inset: TEM images of HSs. c) Absorbance of the resulting $Pb_4S_3Cl_2$ NCs, inset: TEM images of the NCs.

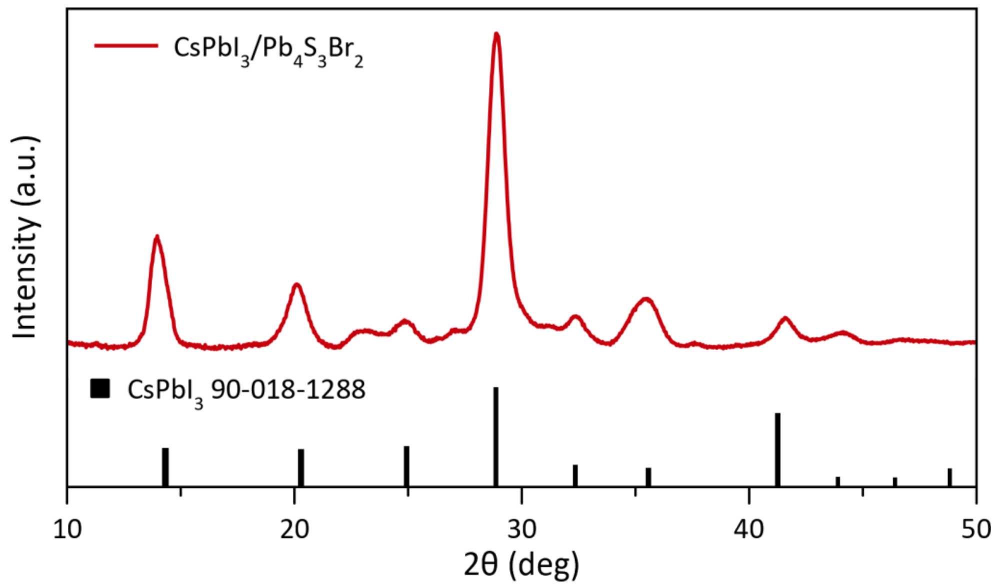

Figure S8. XRD patterns of $CsPbI_3/Pb_4S_3Br_2$ HS. XRD pattern of $CsPbI_3/Pb_4S_3Br_2$ HSs (red) after anion exchange, $CsPbI_3$ reference (black line). The contribution of $Pb_4S_3Br_2$ to the heterostructures pattern is minor due to the much lower volume fraction compared to $CsPbI_3$ and to the spread of diffracted intensities over more peaks due to the lower symmetry.

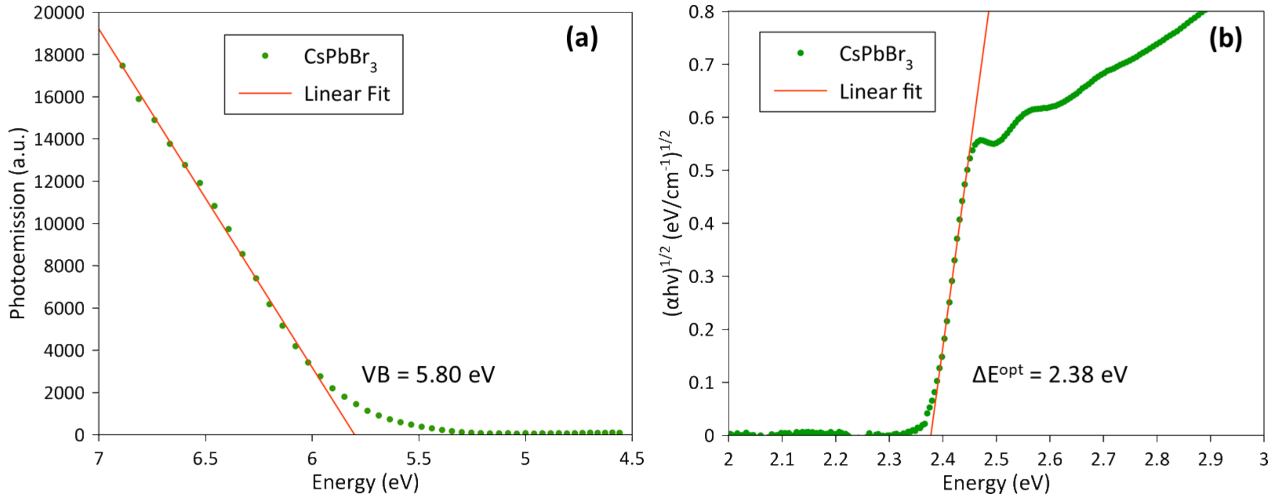

Figure S9. Ambient Pressure Photoemission Spectroscopy and Tauc Plot of $CsPbBr_3$. a) Photoemission of $CsPbBr_3$, used to determine the valence band of the material. b) Tauc plot from the absorbance spectrum of $CsPbBr_3$, used to determine the optical band gap of the material.

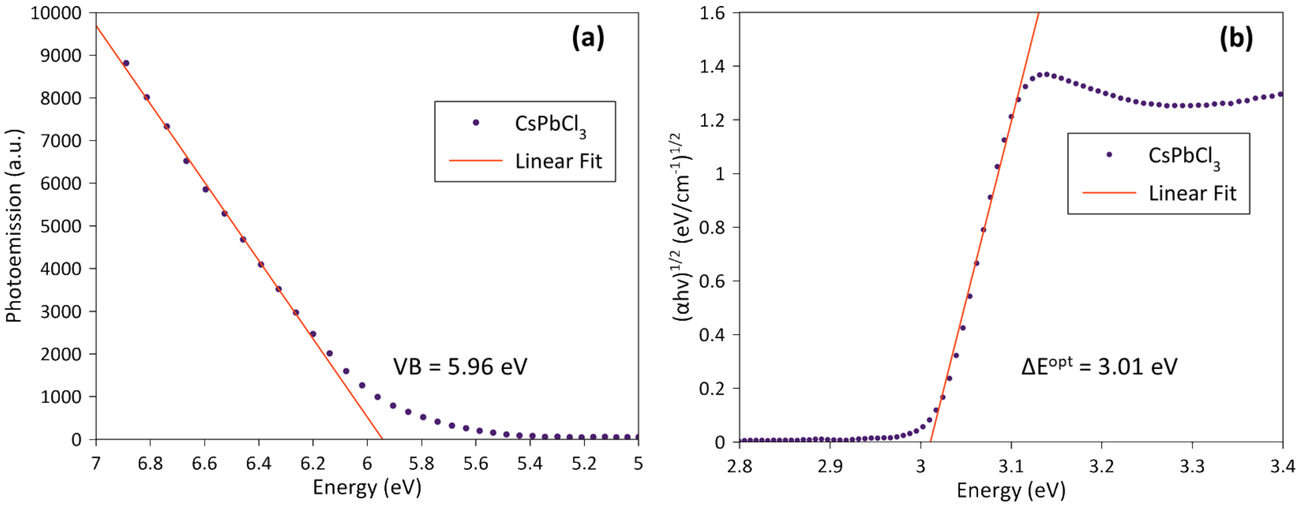

Figure S10. Ambient Pressure Photoemission Spectroscopy and Tauc Plot of $CsPbCl_3$. a) Photoemission of $CsPbCl_3$, used to determine the valence bad of the material. b) Tauc plot from the absorbance spectrum of $CsPbCl_3$, used to determine the optical band gap of the material.

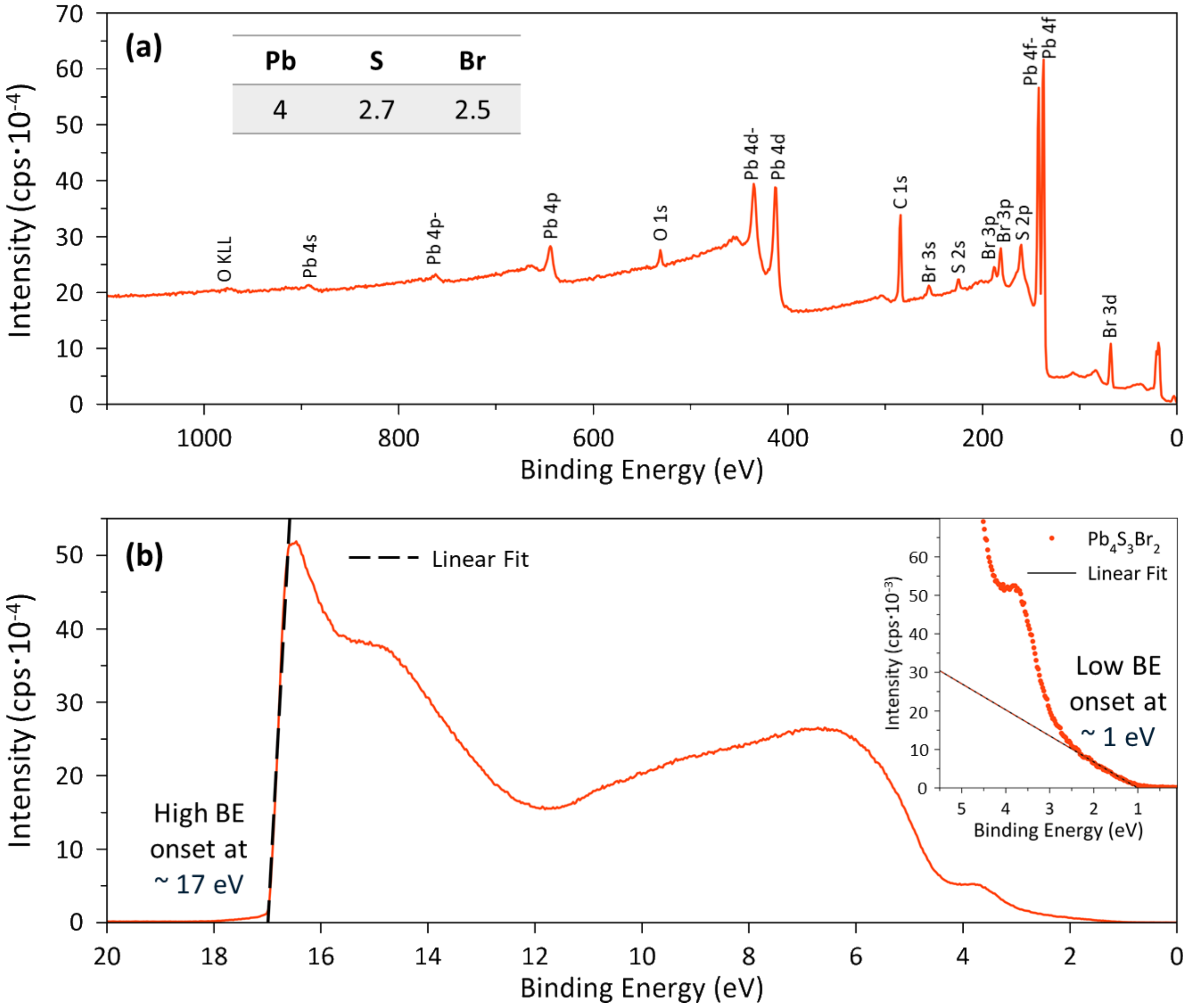

Figure S11. X-ray Photoelectron Spectroscopy (XPS) and Ultraviolet Photoelectron Spectroscopy (UPS). a) XPS data for $Pb_4S_3Br_2$. Inset: composition of element by XPS analysis, in agreement with the stoichiometry of the material. b) UPS data for the valence band of $Pb_4S_3Br_2$ that is found at 1.0 eV ± 0.1 eV eV *vs* Fermi Level, since the work function is 4.3 eV, the valence band vs vacuum level is at 5.23 eV. The high and low binding energy onsets were obtained *via* linear extrapolation and are marked.

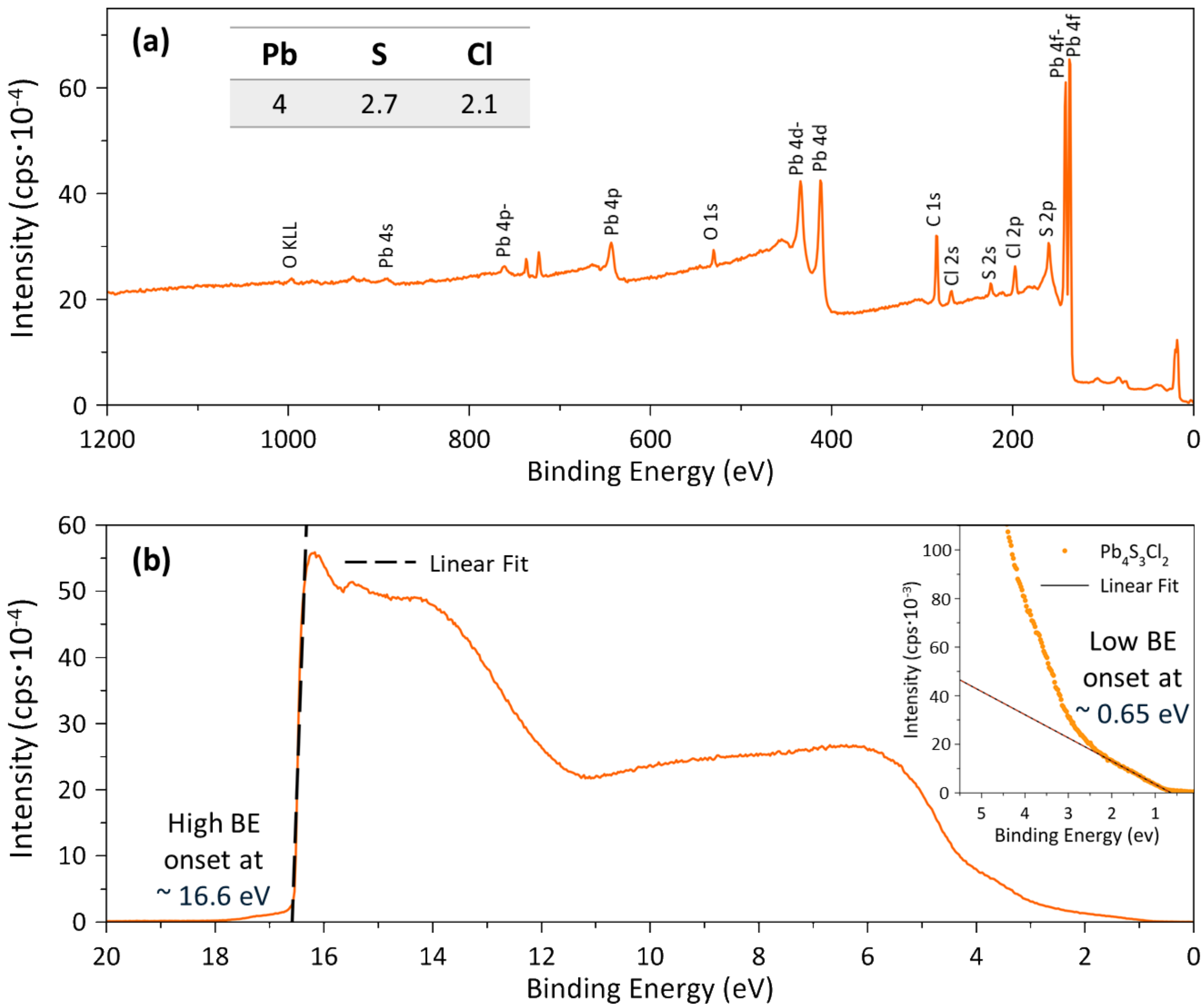

Figure S12. X-ray Photoelectron Spectroscopy (XPS) and Ultraviolet photoelectron Spectroscopy (UPS). a) XPS data for $Pb_4S_3Cl_2$. Inset: composition of elements by XPS analysis, in agreement with the stoichiometry of the material. b) UPS data for the valence band of $Pb_4S_3Cl_2$ that is found at 0.65 eV vs Fermi Level, since the work function is 4.67, eV the valence band vs vacuum level is at 5.32 eV. The high and low binding energy onsets were obtained *via* linear extrapolation and are marked.

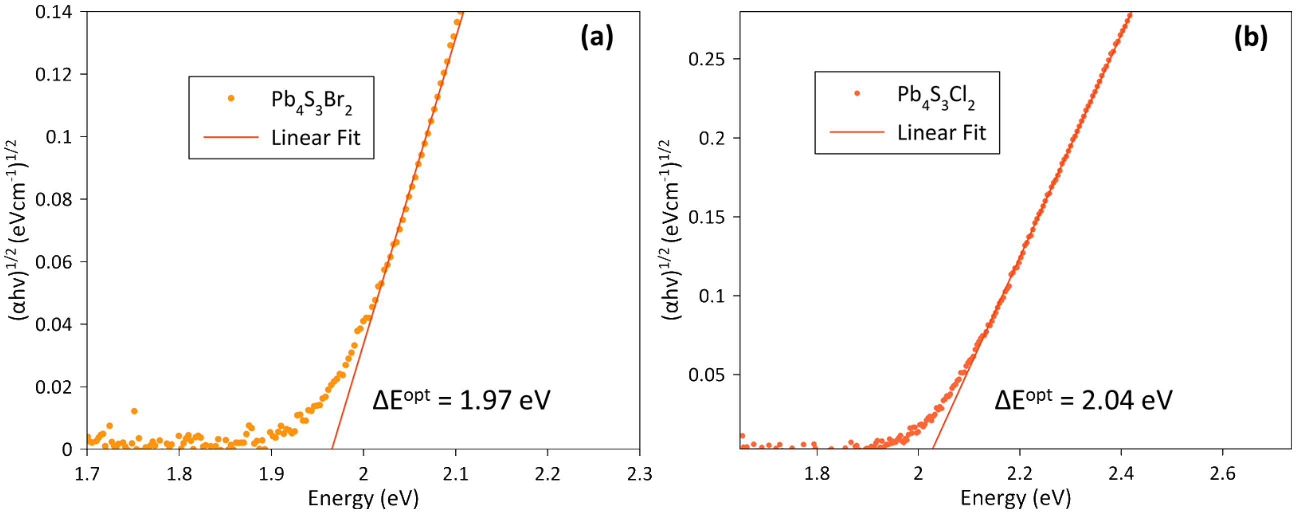

Figure S13. Tauc plots. a) Tauc plot from the absorbance spectrum of $Pb_4S_3Br_2$, used to determine the optical band gap of the material. b) Tauc plot from the absorbance spectrum of $Pb_4S_3Cl_2$, used to determine the optical band gap of the material.

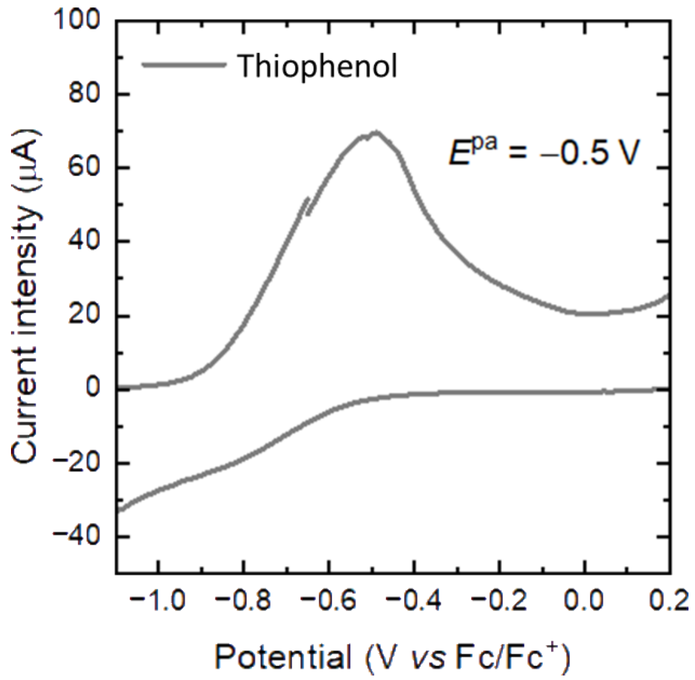

Figure S14. Electrochemical measurements. CV curve of thiophenol, used to determine the highest occupied molecular orbital (HOMO) of the molecule (-4.2 eV) at the anodic peak maximum.

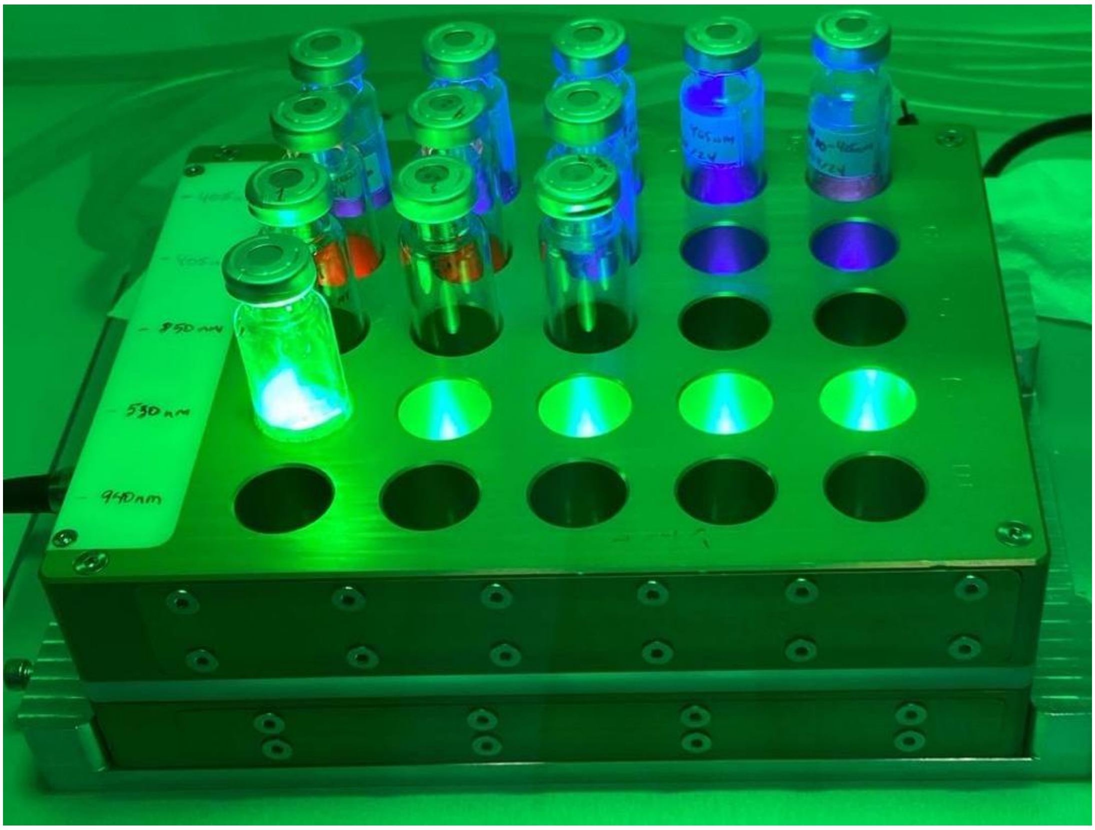

Figure S15. The photoreactor used for the photoreactions is equipped with five LED lines, with different wavelength emissions.

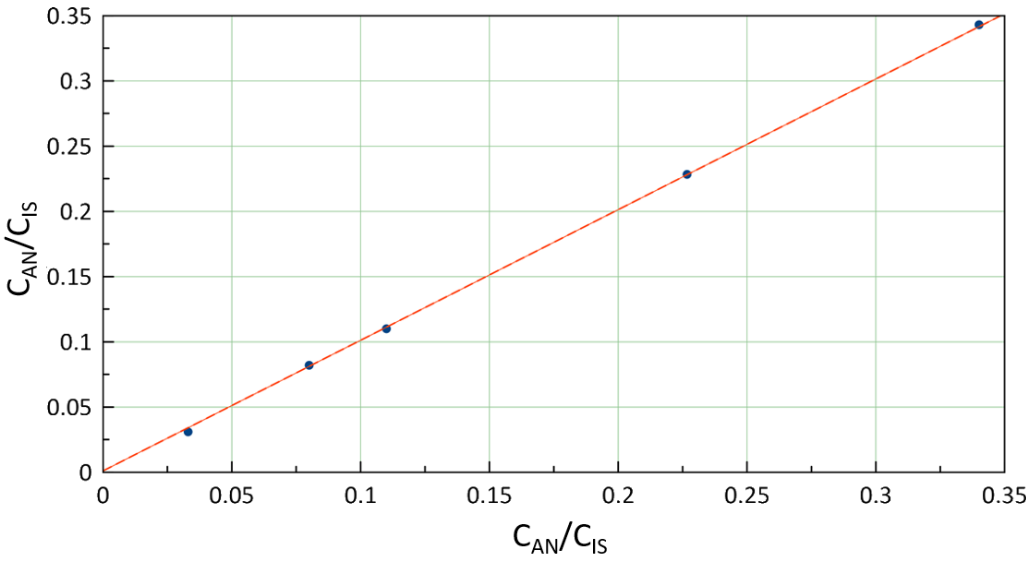

Figure S16. Calibration curve. Correlation between concentration and peak area ratio (analyte/IS).

Table S1. GC retention time of the different compounds.

Retention time (min)	Compound
15.122 – 15.128	Biphenyl (internal standard)
7.627	Thiophenol
20.510	Disulfide diphenyl
26.045	Bis(4-bromophenyl) disulfide
25.454	Bis(4-methoxyphenyl) disulfide
14.015-14.021	Cyclohexanone
19.880-19.887	1-octadecene

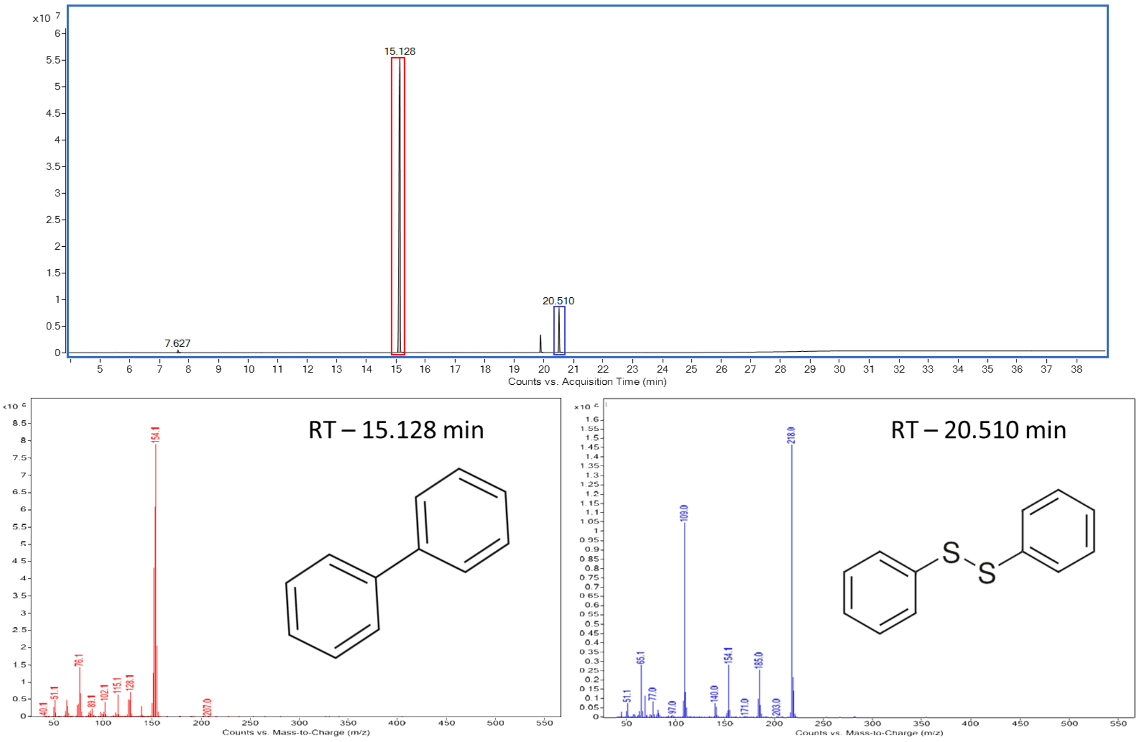

Figure S17. Gas Chromatography – Mass Spectrometry. Gas chromatography of the thiophenol reaction in cyclohexane and mass spectra of the internal standard (bottom left) and the product (bottom right).

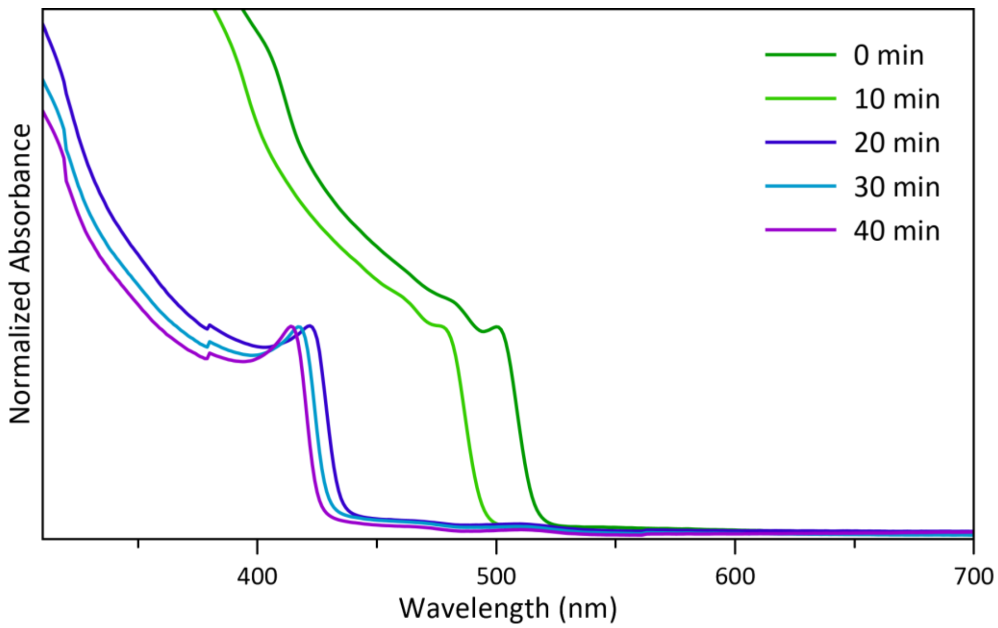

Figure S18. Absorbance spectra of $CsPbBr_3$ in CH_2Cl_2 under light. Time evolution of the absorption spectrum of $CsPbBr_3$ in CH_2Cl_2 under illumination at 450 nm.

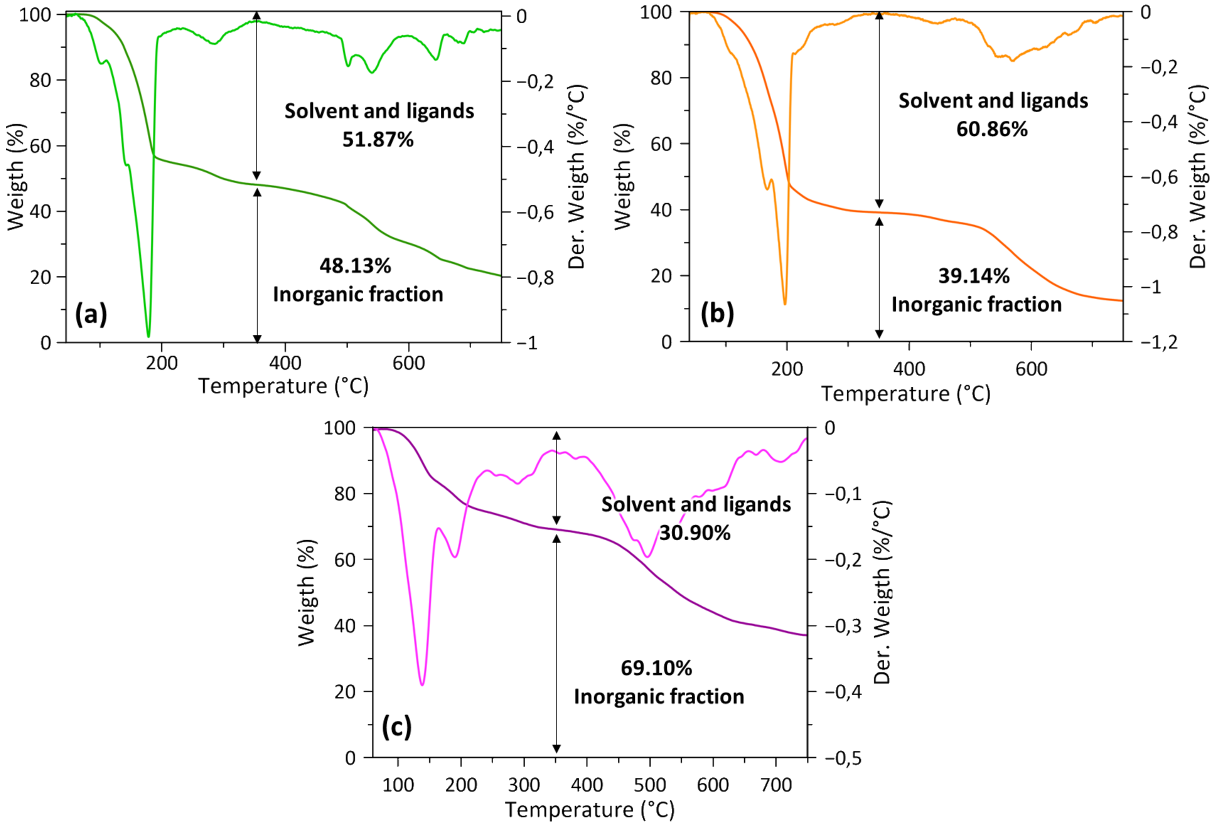

Figure S19. Thermogravimetric analysis. a) TGA analysis of $CsPbBr_3$ NCs (green line) and first derivative (light green line). b) TGA analysis of $CsPbBr_3/Pb_4S_3Br_2$ HSs (orange line) and first derivative (light orange line). c) TGA analysis of $Pb_4S_3Br_2$ NCs (purple line) and first derivative (light purple line). Thermogravimetry analyses (TGA) indicated that the first weight loss is due to two different contributions: hexane adsorbed on the NCs surface, as reported previously,[4] and organic ligands (both bound and free ligands). Therefore, the inorganic NC cores accounted for the remaining 39.1% of weight in the case of the heterostructures, 48.1 % in the case of perovskites, and 69.1 % for the chalcohalides.

Table S2. Comparison of the photocatalytic performances of $CsPbBr_3/Pb_4S_3Br_2$ HSs and other semiconductor photocatalysts for the oxidative coupling of thiophenol under visible light irradiation.

**Photocatalyst**	**Substrate**	**Product yield (%)**	**Selectivity (%)**	**Reaction time**	**Wavelength**	**TON**	**TOF**	**REF**
$CsPbBr_3/Pb_4S_3Br_2$	Thiophenol	81	87	90 min	450 nm	14300	9560	This work
$CsPbBr_3$	Thiophenol	98	not reported	6h	White light	-	-	5
CdS	*p*-OCH_3-PhS	97	99	4h	White light	-	-	6
GR–CdS–Co-Pi composite	*p*-OCH_3-PhS	23 µmol	>99	5h	White light	-	-	7
CdSe/CdS	Thiophenol	87	not reported	2h	415 nm	-	-	8

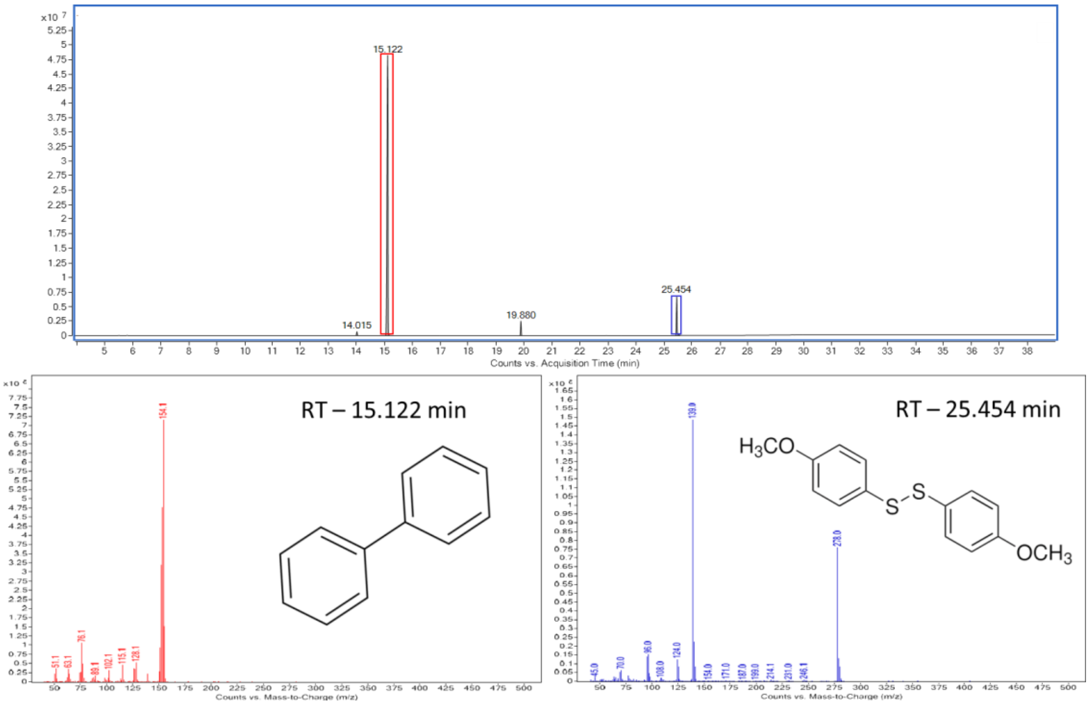

Figure S20. Gas Chromatography – Mass Spectrometry. Gas chromatography of the 4-methoxythiophenol reaction in cyclohexane and mass spectra of the internal standard (bottom left) and the product (bottom right).

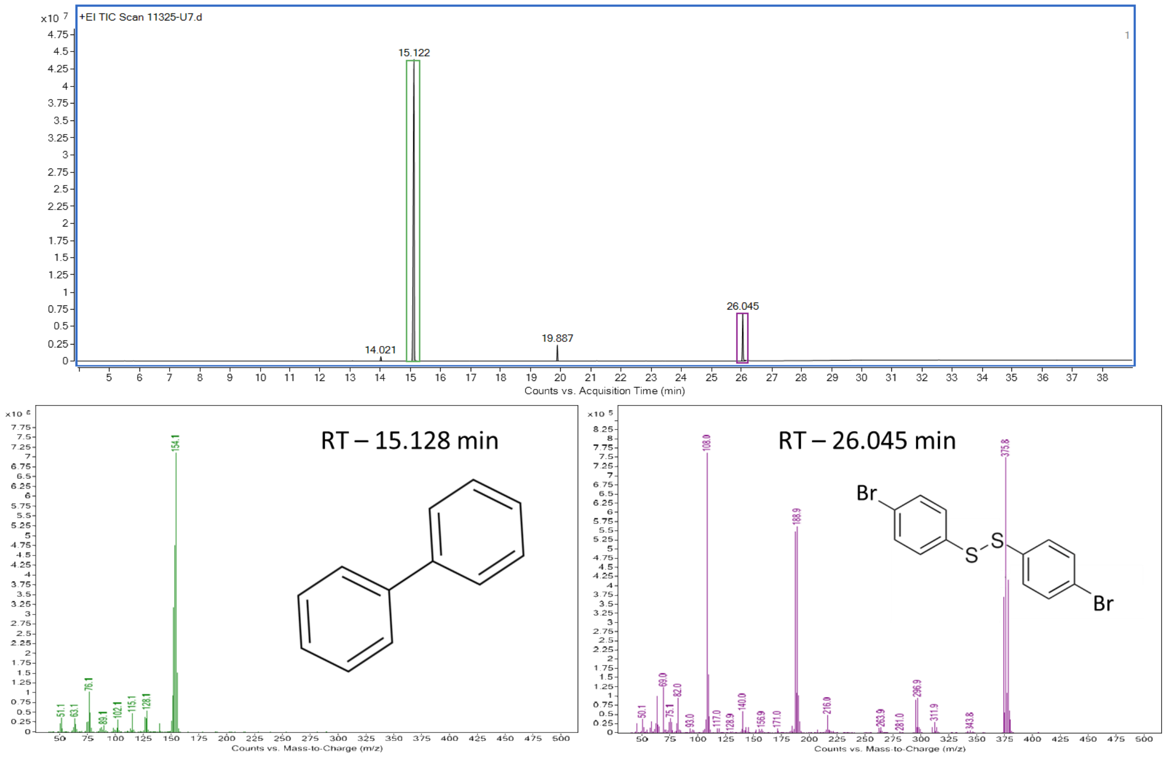

Figure S21. Gas Chromatography – Mass Spectrometry. Gas chromatography of the 4-bromothiophenol reaction in cyclohexane and mass spectra of the internal standard (bottom left) and the product (bottom right).

Table S3. Standard conditions using different scavengers and $CsPbBr_3/Pb_4S_3Br_2$ HS as photocatalyst for the coupling of the thiophenol.

Entry	Scavenger	Variance	Conversion (%)	Product 1b (%)
**1**	1,4 Benzoquinone	None	97	23
**2**	DIPEA	None	99	61
**3**	TEMPO	None	100	3
**4**	TEMPO	N_2	100	3

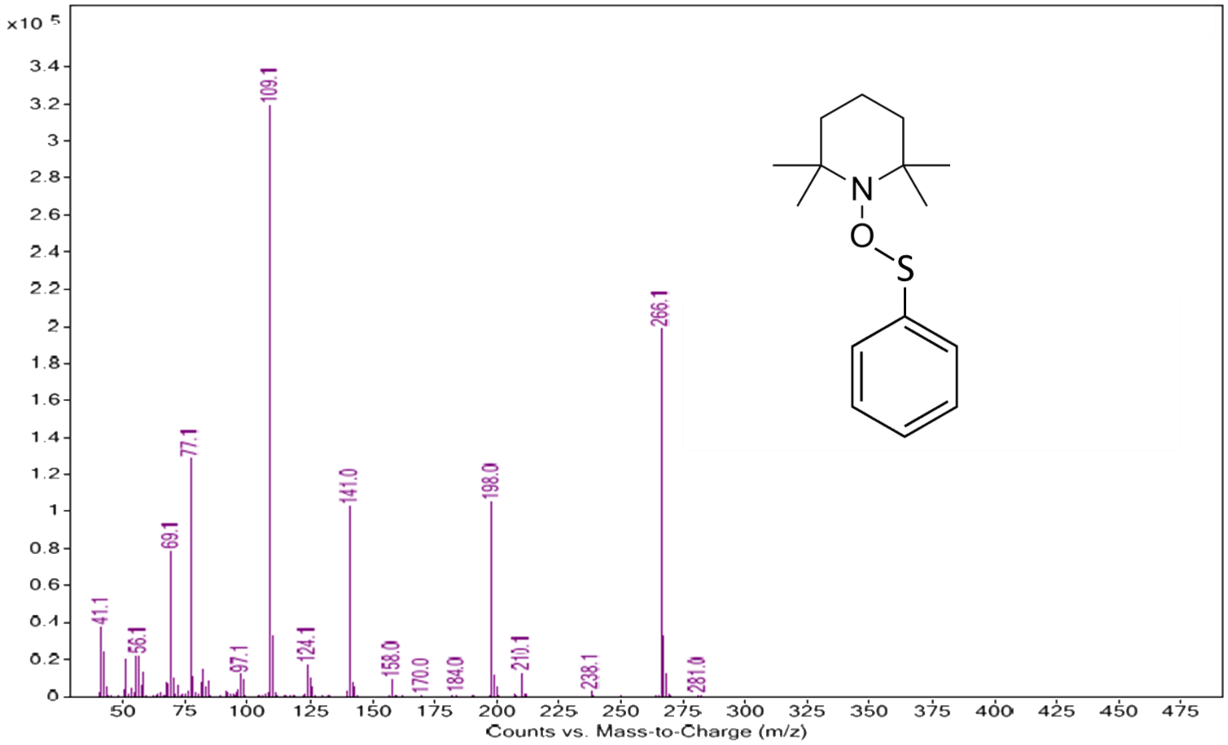

Figure S22. Gas Chromatography – Mass Spectrometry. Mass spectrum of the adduct (TEMPO-PhS) between TEMPO and thiyl radical, showing the typical fragments of both units.

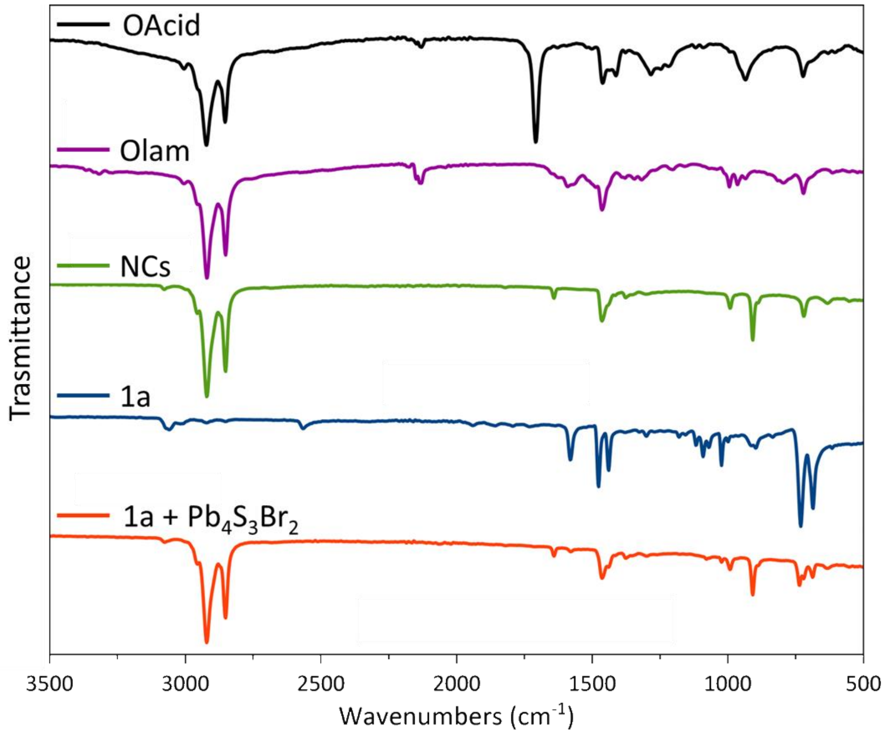

Figure S23. Fourier Transform Infrared Spectroscopy. FTIR spectra of OAcid (black line), Olam (purple line), $Pb_4S_3Br_2$ NCs (green line), 1a-thiophenol (blue line) and a mixture of $Pb_4S_3Br_2$ NCs and 1a (red line).

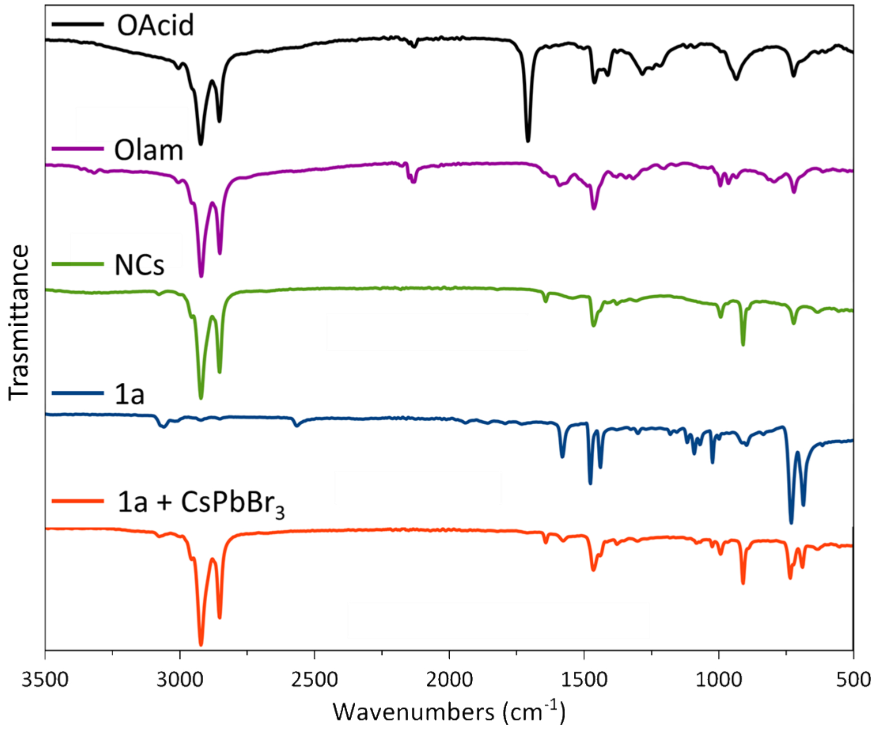

Figure S24. Fourier Transform Infrared Spectroscopy. FTIR spectra of OAcid (black line), Olam (purple line), $CsPbBr_3$ NCs (green line), 1a (thiophenol, blue line) and a mixture of $CsPbBr_3$ NCs and 1a (red line).

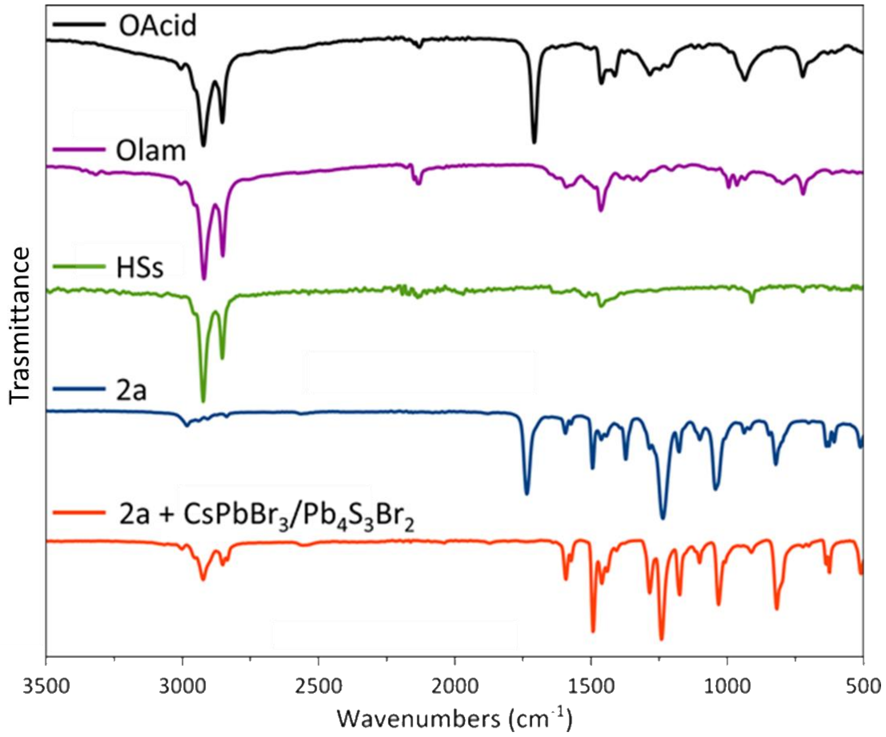

Figure S25. Fourier Transform Infrared Spectroscopy. FTIR spectra of OAcid (black line), Olam (purple line), $CsPbBr_3/Pb_4S_3Br_2$ HSs (green line), 2a (4-methoxythiophenol, blue line) and a mixture of $CsPbBr_3/Pb_4S_3Br_2$ HSs and 2a (red line).

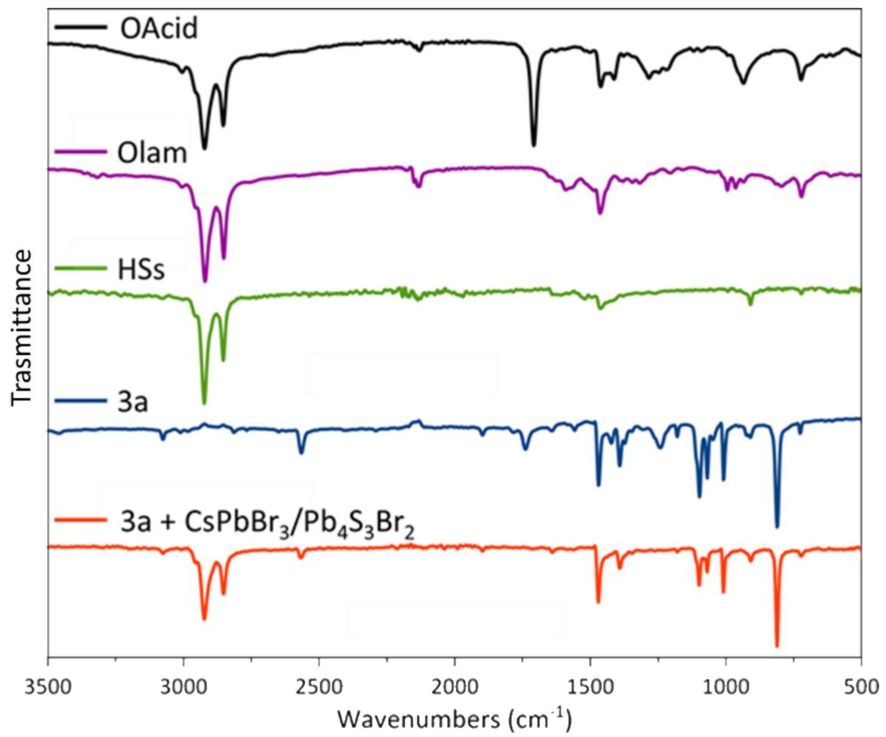

Figure S26. Fourier Transform Infrared Spectroscopy. FTIR spectra of OAcid (black line), Olam (purple line), $CsPbBr_3/Pb_4S_3Br_2$ HSs (green line), 3a (4-bromothiophenol, blue line) and a mixture of $CsPbBr_3/Pb_4S_3Br_2$ HSs and 3a (red line).

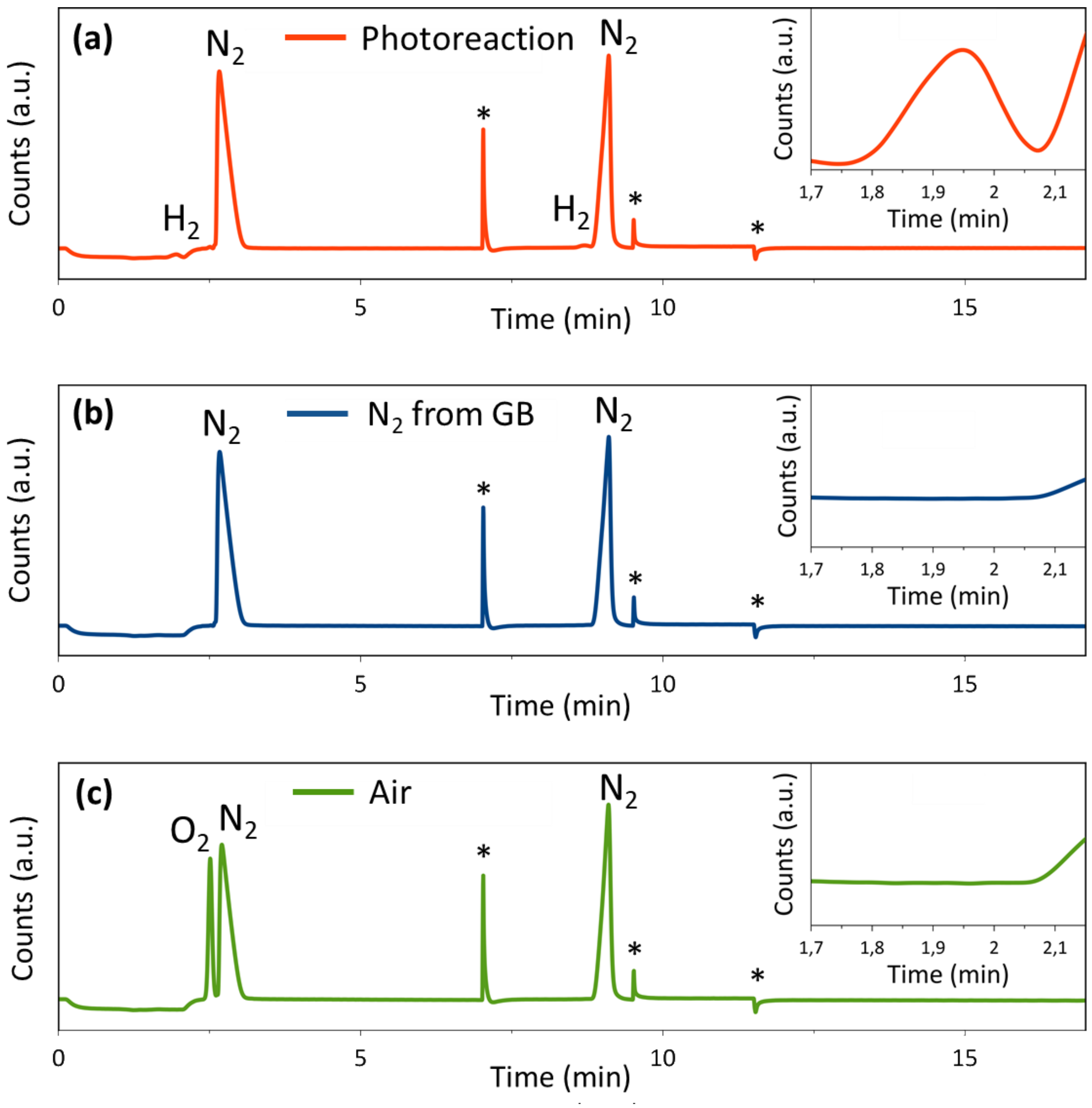

Figure S27. Typical chromatographs (Thermal Conductivity Detector, TCD) obtained upon the injection of controls and photoreaction samples. a) TCD trace of the headspace gas from the photoreaction performed under inert atmosphere (N_2). Inset: magnification of the region corresponding to the first H_2 peak. b) TCD trace of a gas sample collected from the glovebox, showing only N_2. Inset: magnification of the H_2 region, confirming the absence of hydrogen. c) TCD trace of a gas sample taken from air, showing the presence of N_2 and O_2. Inset: magnified view of the H_2 region, again showing no hydrogen signal. Asterisks (*) identify valve events.

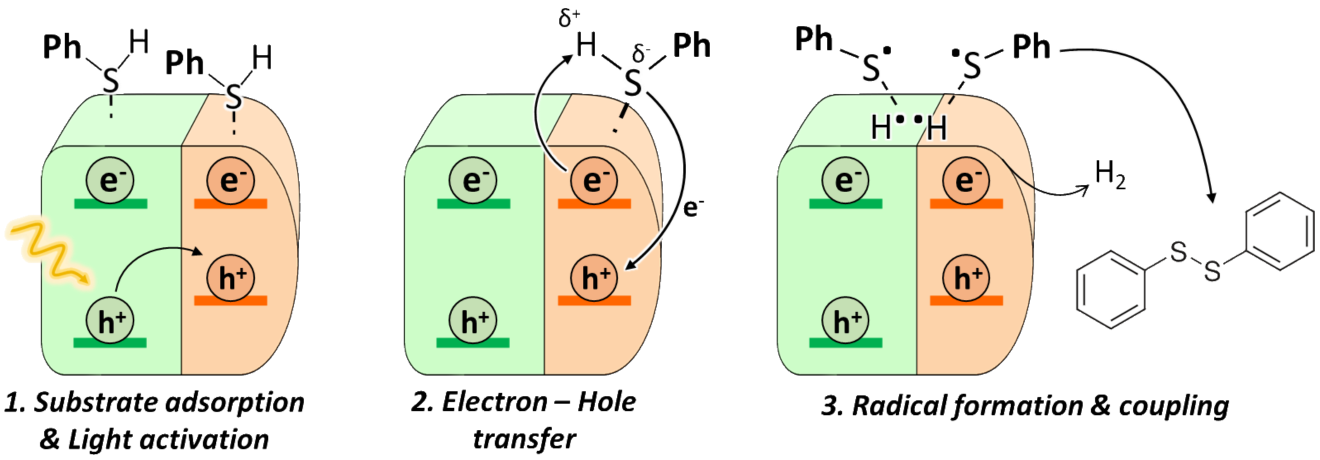

Figure S28. Plausible reaction mechanism in anaerobic conditions. Proposed photocatalytic mechanisms for the oxidative coupling of thiophenol under inert atmosphere, with hydrogen and phenyl disulfide formation.

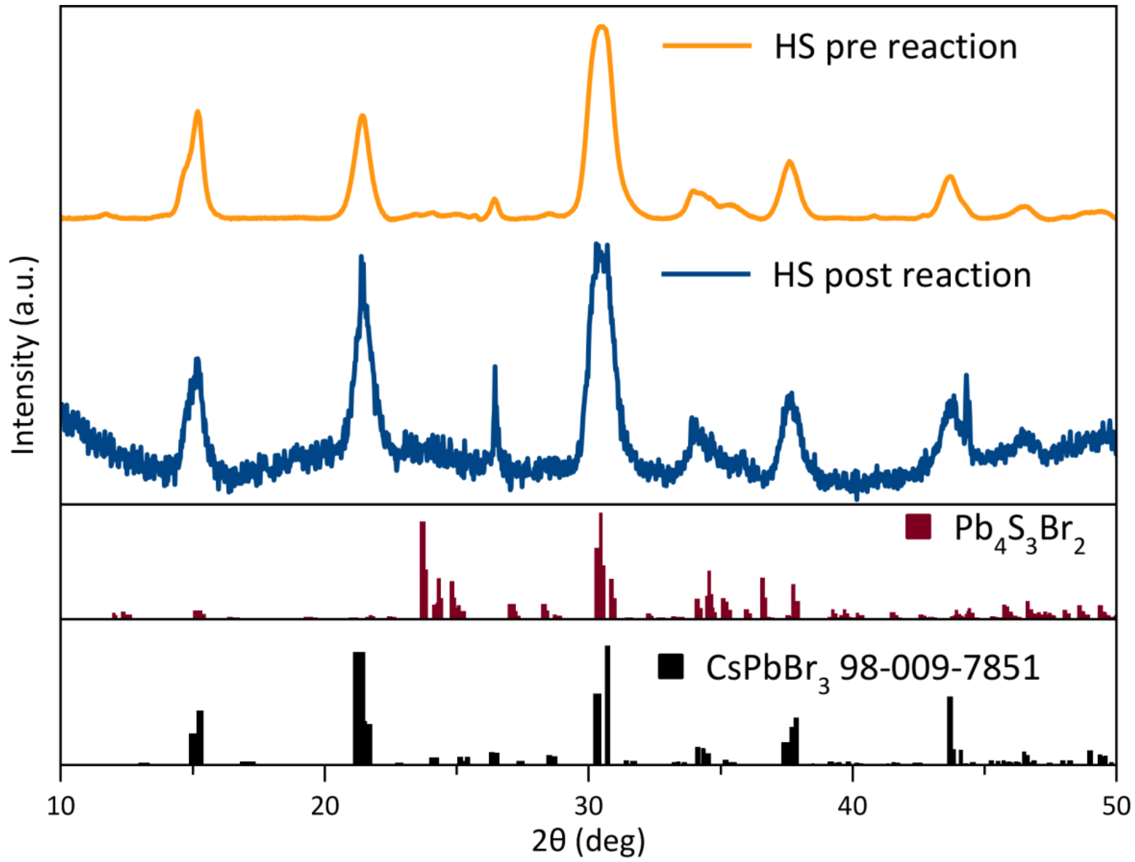

Figure S29. XRD patterns after photoreaction. XRD pattern of $CsPbBr_3/Pb_4S_3Br_2$ HSs before (orange line) and after (blue line) photoreaction and $CsPbBr_3$ (black line), $Pb_4S_3Br_2$ (Bordeaux line) as reference.

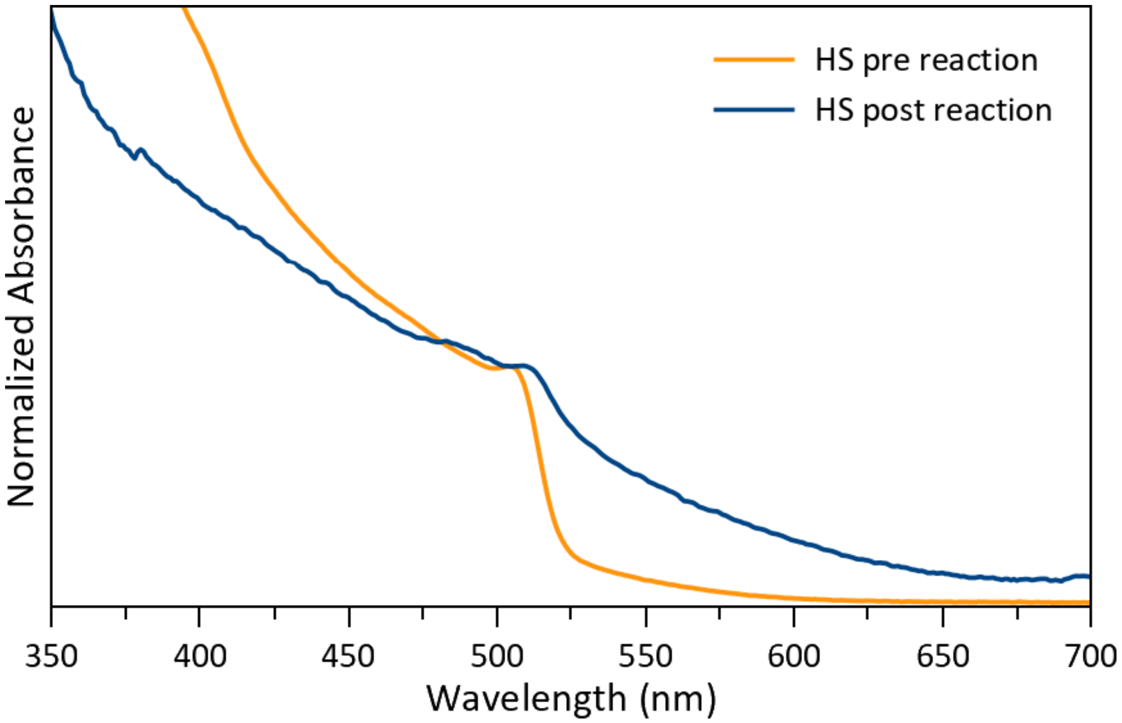

Figure S30. Absorbance spectra after photoreaction. Absorbance spectra of $CsPbBr_3/Pb_4S_3Br_2$ HSs before (orange trace) and after (blue trace) photoreaction.

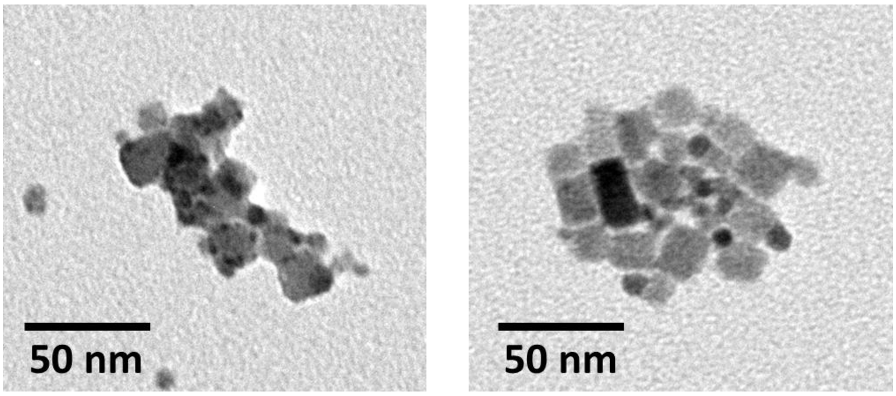

Figure S31. TEM images after photoreaction. TEM images of $CsPbBr_3/Pb_4S_3Br_2$ HSs after photoreaction.

Table S4. Standard conditions using different photocatalysts (Cl-based) for the coupling of the thiophenol and its derivates.

Entry	R substituent	Photocatalyst	Conversion (%)	Product (%)	Selectivity (%)
**1**	-H	$CsPbCl_3/Pb_4S_3Cl_2$	99	70	70
**2**	-H	$CsPbCl_3$	99	47	47
**3**	-H	$Pb_4S_3Cl_2$	73	44	60
**4**	$-OCH_3$	$CsPbCl_3/Pb_4S_3Cl_2$	99	86	86
**5**	$-OCH_3$	$CsPbCl_3$	76	54	71
**6**	$-OCH_3$	$Pb_4S_3Cl_2$	80	52	65
**7**	-Br	$CsPbCl_3/Pb_4S_3Cl_2$	95	77	81
**8**	-Br	$CsPbCl_3$	87	62	71
**9**	-Br	$Pb_4S_3Cl_2$	71	27	38

Table S5. Standard condition using different photocatalysts (I-based) for the coupling of the thiophenol and its derivates.

Entry	R substituent	Photocatalyst	Conversion (%)	Product (%)	Selectivity (%)
**1**	-H	$CsPbI_3/Pb_4S_3Br_2$	100	80	80
**2**	-H	$CsPbI_3$	86	52	61
**3**	$-OCH_3$	$CsPbI_3/Pb_4S_3Br_2$	100	83	83
**4**	$-OCH_3$	$CsPbI_3$	100	82	82
**5**	-Br	$CsPbI_3/Pb_4S_3Br_2$	100	64	64
**6**	-Br	$CsPbI_3$	100	60	60

References

(1) Imran, M.; Peng, L.; Pianetti, A.; Pinchetti, V.; Ramade, J.; Zito, J.; Di Stasio, F.; Buha, J.; Toso, S.; Song, J.; Infante, I.; Bals, S.; Brovelli, S.; Manna, L. Halide Perovskite–Lead Chalcohalide Nanocrystal Heterostructures. *J. Am. Chem. Soc.* **2021**, *143* (3), 1435–1446. https://doi.org/10.1021/jacs.0c10916.
(2) Fairley, N.; Fernandez, V.; Richard-Plouet, M.; Guillot-Deudon, C.; Walton, J.; Smith, E.; Flahaut, D.; Greiner, M.; Biesinger, M.; Tougaard, S.; Morgan, D.; Baltrusaitis, J. Systematic and Collaborative Approach to Problem Solving Using X-Ray Photoelectron Spectroscopy. *Applied Surface Science Advances* **2021**, *5*, 100112. https://doi.org/10.1016/j.apsadv.2021.100112.
(3) Helander, M. G.; Greiner, M. T.; Wang, Z. B.; Lu, Z. H. Pitfalls in Measuring Work Function Using Photoelectron Spectroscopy. *Appl. Surf. Sci.* **2010**, *256* (8), 2602–2605. https://doi.org/10.1016/j.apsusc.2009.11.002.
(4) Ramanath, G.; D'Arcy-Gall, J.; Maddanimath, T.; Ellis, A. V.; Ganesan, P. G.; Goswami, R.; Kumar, A.; Vijayamohanan, K. Templateless Room-Temperature Assembly of Nanowire Networks from Nanoparticles. *Langmuir* **2004**, *20* (13), 5583–5587. https://doi.org/10.1021/la0497649.
(5) Wu, W.-B.; Wong, Y.-C.; Tan, Z.-K.; Wu, J. Photo-Induced Thiol Coupling and C–H Activation Using Nanocrystalline Lead-Halide Perovskite Catalysts. *Catal. Sci. Technol.* **2018**, *8* (16), 4257–4263. https://doi.org/10.1039/C8CY01240G.
(6) Qi, M.-Y.; Shao, X.-N.; Tang, Z.-R.; Xu, Y.-J. not reported (7) Sun, M.-H.; Qi, M.-Y.; Tang, Z.-R.; Xu, Y.-J. Dual Cocatalysts Decorated CdS Nanoparticles for Efficient Dehydrocoupling of Thiols into Disulfides. *Appl. Catal. B Environ.* **2023**, *321*, 122019. https://doi.org/10.1016/j.apcatb.2022.122019.
(8) Sha, Y.; Lin, X.-M.; Niklas, J.; Poluektov, O. G.; Diroll, B. T.; Lin, Y.; Wen, J.; Hood, Z. D.; Lei, A.; Shevchenko, E. V. Insights into the Extraction of Photogenerated Holes from CdSe/CdS Nanorods for Oxidative Organic Catalysis. *J. Mater. Chem. A* **2021**, *9* (21), 12690–12699. https://doi.org/10.1039/D1TA01124C.
